# Genic Intolerance to Functional Variation and the Interpretation of Personal Genomes

**DOI:** 10.1371/journal.pgen.1003709

**Published:** 2013-08-22

**Authors:** Slavé Petrovski, Quanli Wang, Erin L. Heinzen, Andrew S. Allen, David B. Goldstein

**Affiliations:** 1 Center for Human Genome Variation, Duke University, School of Medicine, Durham, North Carolina, United States of America; 2 Departments of Medicine, The University of Melbourne, Austin Health and Royal Melbourne Hospital, Melbourne, Victoria, Australia; 3 Department of Medicine, Section of Medical Genetics, Duke University, School of Medicine, Durham, North Carolina, United States of America; 4 Department of Biostatistics and Bioinformatics, Duke University, Durham, North Carolina, United States of America; Dartmouth College, United States of America

## Abstract

A central challenge in interpreting personal genomes is determining which mutations most likely influence disease. Although progress has been made in scoring the functional impact of individual mutations, the characteristics of the genes in which those mutations are found remain largely unexplored. For example, genes known to carry few common functional variants in healthy individuals may be judged more likely to cause certain kinds of disease than genes known to carry many such variants. Until now, however, it has not been possible to develop a quantitative assessment of how well genes tolerate functional genetic variation on a genome-wide scale. Here we describe an effort that uses sequence data from 6503 whole exome sequences made available by the NHLBI Exome Sequencing Project (ESP). Specifically, we develop an intolerance scoring system that assesses whether genes have relatively more or less functional genetic variation than expected based on the apparently neutral variation found in the gene. To illustrate the utility of this intolerance score, we show that genes responsible for Mendelian diseases are significantly more intolerant to functional genetic variation than genes that do not cause any known disease, but with striking variation in intolerance among genes causing different classes of genetic disease. We conclude by showing that use of an intolerance ranking system can aid in interpreting personal genomes and identifying pathogenic mutations.

## Author Summary

This work uses empirical single nucleotide variant data from the NHLBI Exome Sequencing Project to introduce a genome-wide scoring system that ranks human genes in terms of their intolerance to standing functional genetic variation in the human population. It is often inferred that genes carrying relatively fewer or relatively more common functional variants in healthy individuals may be judged respectively more or less likely to cause certain kinds of disease. We show that this intolerance score correlates remarkably well with genes already known to cause Mendelian diseases (P<10^−26^). Equally striking, however, are the differences in the relationship between standing genetic variation and disease causing genes for different disease types. Considering disorder classes defined by Goh et al (2007) human disease network, we show a nearly opposite pattern for genes linked to developmental disorders and those linked to immunological disorders, with the former being preferentially caused by genes that do not tolerate functional variation and the latter caused by genes with an excess of common functional variation. We conclude by showing that use of an intolerance ranking system can facilitate interpreting personal genomes and can facilitate identifying high impact mutations through the gene in which they occur.

## Introduction

Many approaches are available that attempt to prioritize mutations in terms of their prior probabilities of conferring risk of disease, notably including population allele frequency and measures of conservation at either the phylogenetic level [1] or in terms of amino acid characteristics [2]–[6]. However, few analogous approaches are available for prioritizing the genes in which the variants are found, despite the fact that all groups performing contemporary sequencing studies have learned that some genes are much more likely to show at least modest (but unconvincing) evidence of association with risk across multiple disease areas than other genes. One reason for this outcome is that some genes carry many more putatively interesting variants in the general population, leading to more potential to show association for such variants. Here, we seek to develop a gene-level assessment that ranks genes in terms of their real likelihoods to influence disease.

The basis of our approach is to rank all protein-coding human genes in terms of their intolerance to standing functional variation. This scheme is intended to rank genes on the basis of the strength and consistency of purifying selection acting against functional variation in the gene. We note, however, that any such scheme will inevitably also reflect the action of other kinds of selection (for example, balancing selection). Such a scoring system can be constructed in many ways, but it would need to be standardized for gene size and total mutational rate. Using publically available data from the NHLBI Exome Sequencing Project (ESP) [7] we introduce a scoring system that predicts the expected amount of common functional variation based on the total amount of variation in each gene. The intolerance score itself is a measure of the deviation from this prediction.

We evaluate this scoring system by examining correlations between gene scores and whether genes do or do not cause known Mendelian diseases [8]. We further evaluate how well this approach prioritizes candidate *de novo* mutations identified in patient genomes [9]–[15]. Critical to interpreting personal genomes, we show how our gene-level score can be integrated with well-established variant-level scores to highlight candidate causal mutations.

## Results

To develop a gene-level assessment that ranks genes in terms of their likelihoods to influence disease, we primarily rely on three highly curated public datasets. The ESP6500 dataset is our source for aggregate single nucleotide variant (SNV) sequence data, described elsewhere [7], [16]. The CCDS database was used to define genes based on publically assigned transcripts [17]. Finally, the Online Mendelian Inheritance in Man (OMIM) database was used to assess the utility of the score by correlating the score with whether genes do or do not cause Mendelian diseases [8].

Considering genes assigned a HUGO Gene Nomenclature Committee (HGNC) name, we set the coding boundaries of HGNC genes to the public CCDS transcripts (CCDS release 9, GRCh37.p5), with an extension of two base-pairs at each end of exons to allow for splice acceptor and donor variant annotations. For genes with multiple CCDS transcripts, we merged the corresponding regions into a consensus summary of all CCDS-defined bases for that HGNC gene. Using these CCDS boundaries, we considered only CCDS sites reported with at least 10-fold coverage in the ESP6500 database [7]. We then defined “assessable” genes as HGNC genes with at least 70% of their CCDS covered by an average 10-fold coverage in the ESP6500 database. This resulted in 16,956 assessable HGNC genes with CCDS transcript(s). We adopted the annotated variant effect predictions provided in the ESP6500 database, described elsewhere [16]. We classified missense, nonsense, and splice acceptor/donor variants as “functional,” and synonymous variants as “non-functional,” recognizing that such classifications will never be entirely accurate. The ESP6500 database also includes indel variants, but as these are less accurately called than SNVs, we have excluded them from current analyses [18]. In assessing the utility of the score, we organized Mendelian disease genes on the basis of genetic models, considering the following groups: “haploinsufficient,” “dominant-negative,” “*de novo* disease-causing,” “recessive,” and “non-disease” genes using the OMIM database (accessed 3^rd^ December 2012) (Methods and Dataset S1).

### Deriving the Residual Variation Intolerance Score

**Figure 1:**
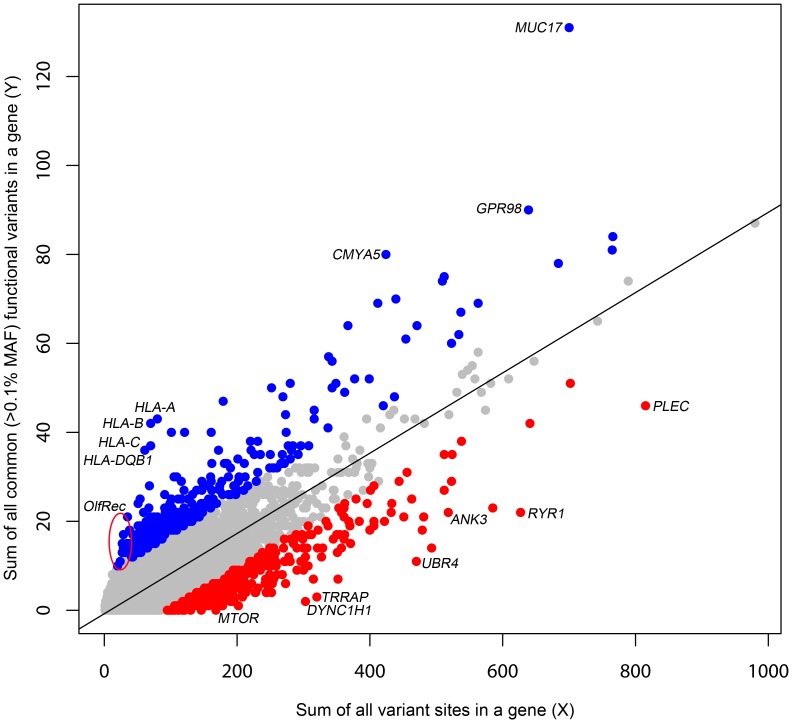
A regression plot illustrating the regression of Y on X. The plot is annotated for the 2% extremes: red = 2% most intolerant, blue = 2% most tolerant. Five outlier genes with >140 common functional variant sites (y-axis) are not shown.

The primary motivation behind a gene based intolerance score is to quantitatively distinguish two categories of genes. On one hand, the *ATP1A3* gene has very few functional mutations in the general population, which makes it all the more striking when 70% of patients with alternating hemiplegia of childhood were found to carry *de novo* missense mutations in the gene [19]. On the other hand, olfactory receptor genes often carry non-conservative amino acid substitutions and stop mutations at high frequencies in human populations yet trigger no clinical diagnosis. Clearly, to suggest causation, it would take more observations of functional mutations in patients in an olfactory receptor gene than in *ATP1A3*. To quantitatively capture this difference, we derive a score, based on the combined ESP6500 dataset that assesses the degree to which genes have either more or less common functional variation than expected for the genome as a whole given the amount of presumably neutral variation they carry. We define the threshold dividing “common” and “rare” variants as ρ. We then define Y as the total number of common (Minor Allele Frequency [MAF]>ρ) missense and “truncating” SNVs (including splice and nonsense variants) and X as the total number of protein-coding variants (including synonymous variants, regardless of frequency in the population) observed within a gene. We then regress Y on X (Figure 1) and take the studentized residual as the Residual Variation Intolerance Score (RVIS). Thus, the raw residual is divided by an estimate of its standard deviation and accounts for differences in variability that come with differing mutational burdens. The RVIS then provides a measure of the departure from the (genome-wide) average number of common functional mutations found in genes with a similar amount of mutational burden. When S = 0, the gene has the average number of common functional variants given its total mutational burden; when S<0, the gene has less common functional variation than predicted; when S>0, it has more. Although multiple population genetic forces could influence the RVIS value of a gene, negative scores are likely to often reflect purifying selection, whereas positive scores are likely to reflect either the absence of purifying selection, the presence of some form of balanced or positive selection, or both. Scores for the 16,956 assessed genes are available in Dataset S2, and a histogram of the distribution of S is available in Figure S1.

Here, we have set ρ = 0.1% MAF in the combined ESP6500 population. However, we also explored the behaviour of the score for ρ of 0.01% and 1%, and found both of these to be strongly correlated with ρ = 0.1% (Pearson's *r* = 0.849 and Pearson's *r* = 0.813, respectively) (Figure S2).

To facilitate interpretation, we also present the RVIS values as percentiles that reflect the relative rank of the genes, with the lowest scores being the most intolerant genes.

### Correlation between RVIS and genes that cause Mendelian disease

The residual variation intolerance score is derived using the combined European American (EA) and African American (AA) data. Detailed studies of the EA and AA data, within the exome sequencing project (ESP), have been published elsewhere [16]. Here, we show that there is a strong correlation between RVIS values based on the combined population compared to scores based on either the EA samples or AA samples: Pearson's *r* = 0.86 and Pearson's *r* = 0.91, respectively (Figure S2). To address whether the RVIS is a predictor of “common” mutations and mutational burden, we also compared a score derived from the EA polymorphism data to the score derived from the AA polymorphism data. These two populations generate two independently derived RVISs for each gene. For the EA versus AA RVIS comparison, the Pearson's *r* correlation is 0.73 (Figure S2 [G]).

**Figure 2:**
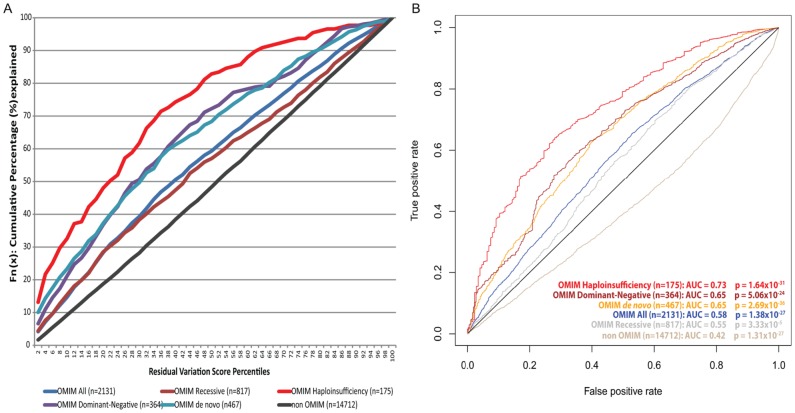
[A] Cumulative percentage plots for the residual variation intolerance scores among six OMIM lists. [B] ROC curves of the residual variation intolerance scores' capacity to predict the corresponding OMIM list.

To assess whether the RVIS can discriminate genes that do and do not cause disease, we compared the RVIS values for genes causing different kinds of Mendelian diseases. Using keyword searches in OMIM, we extracted six gene-lists reflecting different contexts: OMIM genes, “haploinsufficiency,” “dominant-negative,” “*de novo*” disease causing, “recessive,” and we indirectly derived a non-disease gene list (Methods, Table 1, and Dataset S1). Using a logistic regression model, we found that genes causing Mendelian diseases have lower RVIS values than those that do not, with the strongest correlations observed for haploinsufficiency (p = 1.6×10^−31^; *β* = −0.71 [95%CI −0.82–−0.59]) and *de novo* disease-linked events (p = 2.7×10^−36^; *β* = −0.57 [95%CI −0.65–−0.48]) (Table 1, Figure 2). ROC curves were generated to illustrate the capacity of the RVIS to predict the OMIM gene lists (Figure 2B).

**Table 1:** Summarizing the RVIS and RVIS-PP2 performance in settings of Online Mendelian Inheritance in Man (OMIM) and Mouse Genome Informatics (MGI) gene lists.

	OMIM disease genes	“recessive”	“HI”	“dominant negative”	“*de novo*”	“HI” and “*de novo*”	MGI ortholog “lethality”	MGI ortholog “seizure”	Essential Gene List∧
Keyword search	2329*	881	202*	387	507	N/A	99	99	2472
No CCDS transcript	46	15	2	4	10	N/A	1	1	28
<70% NHLBI-ESP gene capture	152	49	25	19	30	N/A	7	3	156
Total (%)	2131 (91.5%)	817 (92.7%)	175 (86.6%)	364 (94.1%)	467 (92.1%)	108	91 (91.9%)	95 (96.0%)	2288 (92.6%)
**RVIS** Significance# [95% CI]	**1.38×10^−27^** *β* = −0.29 [−0.3 – −0.2]	**3.33×10^−5^** *β* = −0.16 [−0.2 – −0.1]	**1.64×10^−31^** *β* = −0.71 [−0.8 – −0.6]	**5.06×10^−24^** *β* = −0.50 [−0.6 – −0.4]	**2.69×10^−36^** β = −0.57 [−0.7 – −0.5]	**1.39×10^−28^** β = −0.77 [−0.9 – −0.6]	**7.57×10^−13^** β = −0.58 [−0.7 – −0.4]	**1.13×10^−12^** β = −0.57 [−0.7 – −0.4]	**1.25×10^−114^** β = −0.63 [−0.7 – −0.4]
**RVIS** AUC [95% CI DeLong [27], [28]]	**0.58 [0.57–0.59]**	**0.55 [0.53–0.57]**	**0.73 [0.70–0.77]**	**0.65 [0.62–0.68]**	**0.65 [0.62–0.67]**	**0.78 [0.74–0.82]**	**0.69 [0.63–0.75]**	**0.70 [0.65–0.75]**	**0.66 [0.65–0.67]**
**RVIS** Mann-Whitney U **	**2.02×10^−32^**	**1.37×10^−8^**	**1.02×10^−27^**	**1.89×10^−24^**	**2.16×10^−29^**	**1.36×10^−24^**	**6.86×10^−11^**	**2.49×10^−12^**	**1.05×10^−136^**
**RVIS-PP2 version** Significance# [95% CI]	**1.38×10^−29^** *β* = −0.33 [−0.4 – −0.3]	**4.65×10^−7^** *β* = −0.22 [−0.3 – −0.1]	**3.36×10^−20^** *β* = −0.63 [−0.8 – −0.5]	**6.75×10^−10^** *β* = −0.37 [−0.5 – −0.3]	**2.69×10^−25^** β = −0.52 [−0.6 – −0.4]	**5.11×10^−16^** β = −0.66 [−0.8 – −0.5]	**1.15×10^−11^** β = −0.61 [−0.8 – −0.4]	**1.01×10^−7^** β = −0.52 [−0.7 – −0.3]	**4.87×10^−80^** β = −0.56 [−0.6 – −0.5]
**RVIS-PP2 version** AUC [95% CI DeLong [27], [28]]	**0.58 [0.57–0.59]**	**0.55 [0.53–0.57]**	**0.67 [0.63–0.71]**	**0.63 [0.60–0.66]**	**0.62 [0.60–0.65]**	**0.69 [0.64–0.74]**	**0.69 [0.64–0.75]**	**0.66 [0.61–0.71]**	**0.64 [0.63–0.65]**

∧ *Essential gene list was extracted from Georgi et al (2013)* [*21*] *. Described in* *Methods*.

* *OMIM disease and “haploinsufficient (HI)” gene lists were further filtered. Described in* *Methods*.

# *To obtain the presented levels of significance, we used a logistic regression model to regress the presence or absence of a gene, within the corresponding gene list, on the residual variation intolerance score. We provide the corresponding RVIS beta coefficient and its 95% CI*.

** *A Mann-Whitney U test comparing the RVIS of the corresponding gene list to the remaining non OMIM list genes (n = 14,712 genes). Non OMIM control list was revised For MGI lethality (n = 14,672), MGI seizure (n = 14,660), and Essential gene list (n = 13,179), to exclude genes overlapping with corresponding gene list*.

**Figure 3:**
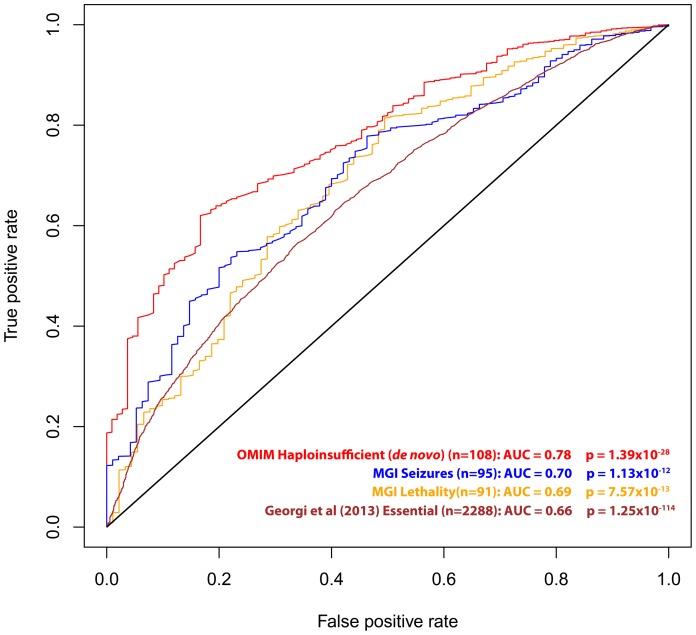
ROC curves of the residual variation intolerance scores' capacity to predict the corresponding independent gene-list.

We also investigated RVIS values for other gene lists of interest, including 91 genes that are human orthologs of “lethality” genes from the Mouse Genome Informatics (MGI) database [20] (mouse knockouts associated with embryonic [MP:0008762], prenatal, [MP:0002080] or perinatal [MP:0002081] lethality), 95 human orthologs of “seizure” genes (mouse knockouts associated with seizures phenotype [MP:0002064]), a set of genes identified as essential in a recent publication by Georgi and colleagues (2013) [21], and the 108 OMIM “haploinsufficiency” genes with *de novo* mutation variants reported (Table 1, Figure 3 and Dataset S1).

We then explored a derivative of the RVIS that is further informed, among the missense mutations, by PolyPhen-2 [2] qualitative predictions (RVIS-PP2). In summary, RVIS-PP2 considers the PolyPhen-2 “benign” classifications as “non-functional” variants (Methods, Table 1, Figure S3). On average, based on the 6503 individuals in the NHLBI-ESP, applying this PolyPhen-2 filter resulted in a 33% reduction of missense variants in any given gene. The RVIS-PP2 values achieved a Pearson's correlation of 0.76 with the RVIS and remain significant across the OMIM disease groups (Table 1).

In part, as the RVIS values reflect the selection pressures acting on genes, one obvious question is the extent to which the RVIS correlates with other measures of selection on genes. One phylogenetic approach is to compare non-synonymous substitutions per non-synonymous site (*d_N_*) to the synonymous substitutions per synonymous site (*d_S_*), as reflected in *ω (aka K_a_/K_s_, d_N_/d_S_)*. To determine whether the RVIS correlates with *ω*, we compared a subset of the genome (the orthologs between human and chimp for human chromosomes 1–5) to three estimates drawn from a separate study (codeml [22], LWL [23], and NG [24]; estimates of *ω* were kindly provided by Dr. Chuanzhu Fan) [25] (Methods). Using a Pearson's *r* correlation, we find that the RVIS is not strongly correlated with these three estimates of *ω*: codeml (*r* = 0.11), LWL (*r* = 0.02), and NG (*r* = 0.04). Moreover, the capacity for the estimates of *ω* to predict OMIM disease genes is inferior to that of RVIS across all investigated gene lists (Table S1 and Figure S4).

### Reviewing disorder classes from the human disease network

**Figure 4:**
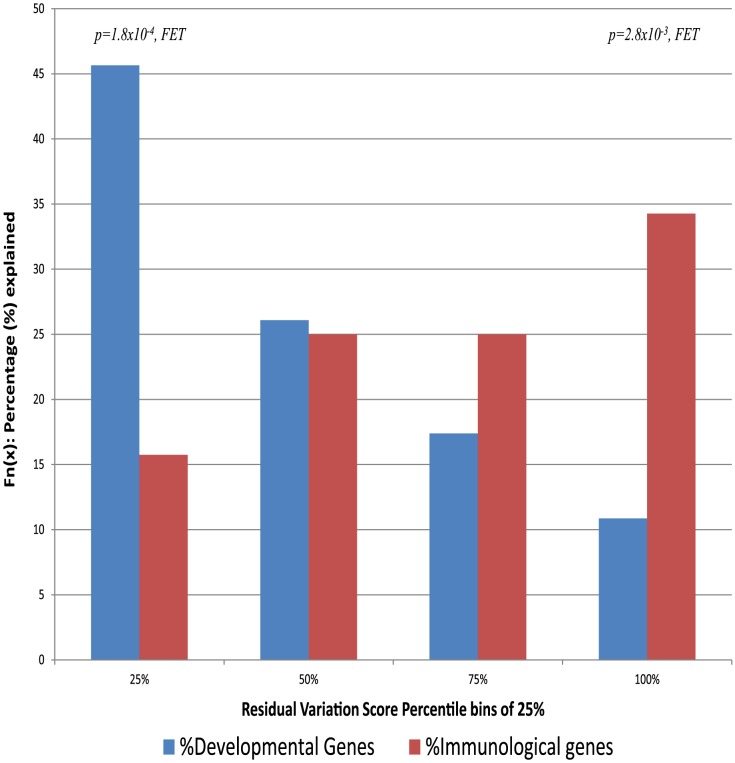
The proportion of genes explained by each of the 25-percentile bins (RVIS) for the human disease networks disorder class with the lowest “Developmental Disorders” and highest “Immunological Disorders” average residual variation intolerance score.

These analyses suggest that genes that are intolerant to genetic variation in the human population are more likely to cause some disorders than genes that either tolerate functional variation or have been under some form of selection promoting functional variation. It remains possible that some kinds of diseases show a different pattern from this overall one. To investigate this possibility we directly assess the gene-lists that make up the 22 disorder classes defined by Goh et al. (2007) [26]. For each disorder class, we assess the average RVIS values (Table S2). This analysis shows striking variation among types of disorders. Some closely follow the overall pattern of being influenced primarily by genes intolerant to functional variation, including “developmental” disorders with an average RVIS of −0.56 (corresponding to 19.54 percentile), “cardiovascular” at −0.45 (corresponding to 24.00 percentile), and “skeletal” at −0.36 (corresponding to 28.64 percentile). At the other extreme there are some disorder classes where it is precisely the genes most enriched in common functional variation that are most likely to cause disease (Table S2). This contrast is illustrated starkly by comparing the two disorder classes with the highest and lowest average RVIS values: developmental diseases and immunological diseases, where we observe that the genes linked to the immunological disorder class have significantly greater tolerance to standing functional variation (Figure 4, *p* = 1.4×10^−5^, 2-tail Mann-Whitney U test). In the former category, approximately half of all OMIM genes causing developmental disorders are found among the genes within the 25^th^ percentile of intolerance and only 10% are found among genes above the 75^th^ percentile. The pattern for immunological disorder OMIM genes is essentially the reverse: only 16% are found among the most intolerant 25^th^ percentile, and 35% above the 75^th^ percentile.

One obvious question is whether genes that cause early onset diseases tend to have lower RVIS values than genes that cause later onset diseases. This is not easy to assess overall, especially given that there are sharp differences in the distributions of ages of onset of diseases in the different categories considered above, and also that not only age of onset but mode of inheritance will influence RVIS (Figure 2). However, to at least partially assess this question, we consider epileptic encephalopathies (EE) and amyotrophic lateral sclerosis (ALS) as two diseases with sharply different ages of onset. We then exclude all EE and ALS OMIM genes reporting only recessive forms (Methods). Of the 10 EE genes linked to a dominant mutation model, the average RVIS = −1.41 (corresponding to the 4.1% most intolerant genes). Of the 13 ALS genes linked to a dominant mutation model, the average RVIS = −0.29 (corresponding to the 33.3% most intolerant genes). Thus, we have described two clearly genetic disorders, differing in age of onset, with an upwards shift in the RVIS corresponding to a later-onset. These analyses suggest that the use of the RVIS values should be tailored, wherever possible, to the RVIS values for genes already securely implicated in the phenotype under study. Focusing on the 25^th^ percentile intolerant genes helped the Epi4K consortium successfully adopt the RVIS to identify epileptic encephalopathy genes within their *de novo* mutation data [15].

### Applying the Residual Variation Intolerance Score to prioritize candidate mutations

So far we have demonstrated the utility of the RVIS to discriminate between OMIM disease genes, and also the disease-causing genes specific to various physiological systems. A recent Epi4K trio sequencing paper illustrated the value of the RVIS in interpreting the *de novo* mutation data from a cohort of sequenced epileptic encephalopathy trios [15]. Here, we show how the residual variation intolerance scores can facilitate the analysis of *de novo* mutations observed in patient genomes.

**Figure 5:**
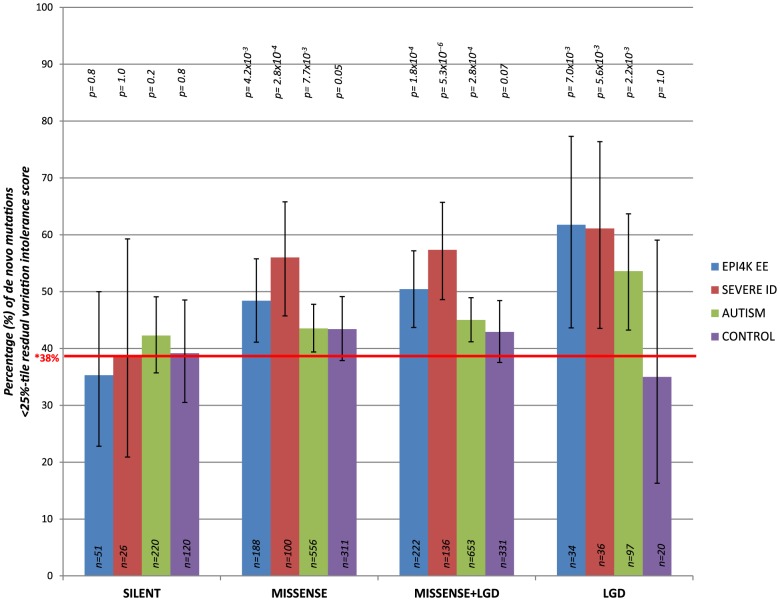
The percentage of *de novo* mutations occurring in the most intolerant quartile (25^th^ percentile) across the severe ID, autistic, epileptic encephalopathy, and control siblings, for the different variant effect types. LGD = Likely Gene Disrupting (including nonsense, coding indels, and splice acceptor/donor site mutations). *Taking the CCDS of RVIS genes, 38% reflects the total real estate occupied by the 25^th^ percentile most intolerant genes. P-values reflect binomial exact tests where the probability of success is adjusted to 0.38, accounting for the gene sizes of the 25% most intolerant genes.

We consider *de novo* mutations observed in patients with severe intellectual disability (ID), epileptic encephalopathies (EE), and autism spectrum disorders (ASD), as well as in control individuals (unaffected siblings that were sequenced across the studies) [9]–[15] (Table S3). Focusing on the 4,264 genes in the most intolerant 25^th^ percentile of RVIS values (Figure S1), we observe an increasing enrichment among intolerant genes for the more extreme mutations (Figure 5 and Table S3). Synonymous *de novo* mutations show no enrichment for intolerant genes in any of the datasets (Figure 5).

Taking the pooled synonymous data across all cohorts (n = 417 synonymous *de novo* mutations) and correcting for the four tests performed, we observe that the functional mutations (missense and likely gene disrupting [LGD]) in the severe ID cohort are significantly enriched for more intolerant genes (p = 1×10^−4^, 2-tail Mann-Whitney U test). Similarly, comparing the EE and ASD cohorts reflect enrichment of likely functional *de novo* mutations preferentially occurring among the most intolerant genes (p = 6.8×10^−3^ and p = 1.3×10^−2^, respectively) (Methods and Table S3). We observe no significance among the functional *de novo* mutations within the control samples, p = 0.12, 2-tail Mann-Whitney U test. Thus, the excess of functional *de novo* mutations observed in intolerant genes among the cohorts ascertained for disease is difficult to explain unless some of those *de novo* mutations actually increase risk of disease.

**Figure 6:**
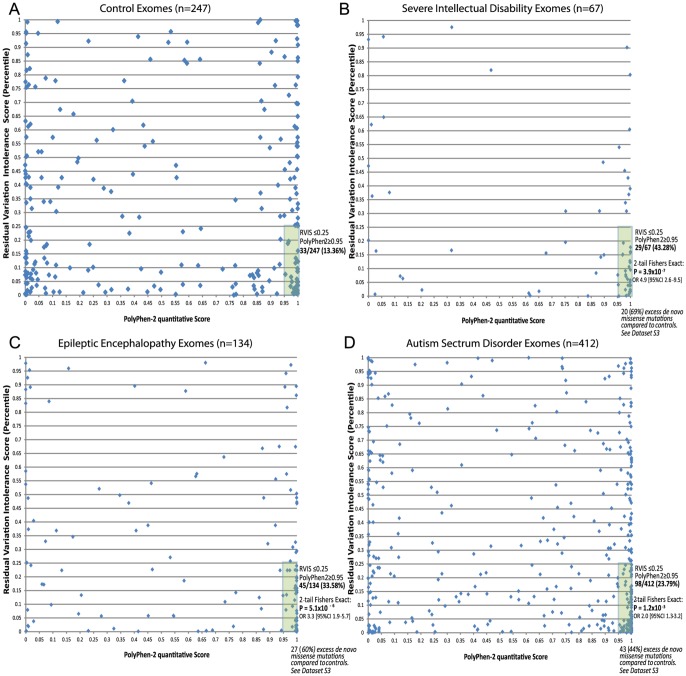
2D plots illustrating possible utility of RVIS in conjunction with a variant-level quantitative score (PolyPhen-2) across cohorts with proposed *de novo* mutation genetic architectures. Plots reflect the single most damaging *de novo* missense mutation in individuals with at least one *de novo* missense mutation: [A] Controls (n = 247); [B] Severe ID (n = 67); [C] Epileptic Encephalopathies (n = 134); [D] Autism Spectrum Disorders (n = 412). Full lists of missense *de novo* mutations in the “hot zone” are available in Dataset S3, including loss of function SNV mutations (not plotted).

The above analyses suggest that gene-level information reflected in the RVIS values can help discriminate between genes that do and do not cause disease. Given the well-established literature that prioritizes variants for their likely pathogenicity, a natural question arises as to whether integrating gene- and variant-level information can improve our ability to pinpoint causal mutations. As the simplest possible illustration of an integrated scheme, we consider two-dimensional (2D) analyses that use the RVIS percentiles for genes (y-axis) and Polyphen-2 quantitative scores for missense mutations (x-axis). We then analysed missense *de novo* mutations observed in the ID, EE, and ASD studies referenced earlier [9]–[15]. We found that, compared to those of controls, *de novo* mutations seen in the exomes of patients showed a striking concentration of density among the most damaging region of the 2D space (Figure 6 [A–D]). A simple interpretation of these data is that while in the general population *de novo* mutations can occur in intolerant genes, and putatively “damaging” *de novo* mutations can occur in the exome, it is much less common for damaging mutations to occur in the most intolerant genes, unless those mutations are contributing to disease. In particular, concentrating only on the lower right-hand-side (y< = 0.25, x> = 0.95), we found that the severe ID (Figure 6B) and EE (Figure 6C) missense *de novo* mutations had a significant excess p = 3.9×10^−7^ and p = 5.1×10^−6^, respectively, compared to control exomes (Figure 6A), and significant, but less enriched, for ASD missense *de novo* mutations (p = 1.2×10^−3^) (Figure 6D and Dataset S3).

## Discussion

The residual variation intolerance score has obvious implications for human disease gene discovery. Of particular relevance is quantifying gene intolerance to functional mutations, genome-wide. Qualitatively, at least for some categories of disease, the genes most likely to influence disease are those that are the most intolerant of functional variation in the human population. More generally, ranking genes based on their RVIS values will clearly help in developing more formal quantitative frameworks that assign weights to genes based on RVIS or elaborations of RVIS.

Several directions for future research could lead to improved gene-based intolerance scoring systems. As both the amount of sequence data and our knowledge of different functional domains of proteins increase, intolerance scoring systems can be developed that subdivide genes based on protein domains as opposed to single gene units. Such approaches could be informative, as certain regions of the gene could be much more constrained than others. Another future direction could be to leverage information from the entire site frequency spectrum (SFS) of mutations within a gene, instead of focusing on functional variation above a given frequency threshold. A gene-based score that incorporated the shifts in the SFS between functional and non-functional variants could produce a more sensitive discriminator of gene intolerance. To better discriminate the putatively non-functional from the functional missense mutations, yet another future direction could be to incorporate variant-level information in the form of conservation scores (e.g. GERP++) [1] or *in silico* protein-damaging characterizing tools (e.g. PolyPhen-2 [2] or SIFT [4]), as we briefly explored in this paper with the RVIS-PP2. A slightly different approach would be to leverage from both a gene-level (RVIS) and a variant-level (e.g., PolyPhen-2) score in prioritizing individual mutations. Initial data (Figure 6) indicate that this approach is particularly promising. Importantly, we have shown that to prioritize causal variants, incorporating both gene- and variant-level information has a demonstrated ability to improve our interpretation of personal genomes.

## Methods

### Estimating coverage-corrected gene-size

We first determine exactly what portion of the whole genome real estate any given gene covers in the ESP6500 database. This step required three parameters:

Coding-sequence source: We adopt the CCDS public transcripts as our coding-sequence source data (CCDS Release 9, Assembly GRCh37.p5), further extending exonic positions by two base pairs, either side of an exon, to permit inclusion of putative splice acceptor and donor sites. For HGNC genes with multiple CCDS transcripts, we merge all transcripts of that gene into a single CCDS boundary. This allows assessment of the overall possible functional burden, correcting for variant annotations based on multiple public CCDS transcripts of HGNC genes.

EVS Ethnicity: The ESP6500 database provides information for variants based on European American (EA), African American (AA), or combined (ALL). For assessing gene intolerance to standing functional variation we adopt the combined (ALL) data. But further compare those results to the EA and AA data (Figure S2).

Minimum Average Coverage: We adopt a minimum average coverage of at least 10-fold for any given CCDS site in the ESP6500 dataset for that site to contribute to assessment of intolerance.

With the above three parameters we extracted data from ESP6500 for each HGNC gene with at least a single public CCDS transcript, including the number of possible sites within the CCDS after the splice acceptor and donor adjustment. We then determined how many of those CCDS defined sites for the HGNC gene had at least 10-fold coverage within the ESP6500 database.

Of the 18,474 gene ids available in CCDS Release 9, 1,518 (8.2%) of genes were determine un-assessable due to having either less than 70% of the possible CCDS covered with at least 10-fold coverage in the ESP6500 database, or, for not having a “public” transcript within CCDS Release 9. This resulted in 16,956 assessable HGNC genes.

### Filtering qualifying variants

We only consider ESP6500 single nucleotide variants (SNV) with a “PASS” filter status, as described on the Exome Variant Server (http://evs.gs.washington.edu/EVS/HelpDescriptions.jsp?tab=tabs-1#FilterStatus; last accessed 12^th^ December 2012).

Variant Function: The coding variant annotations considered for CCDS defined sites include: “missense”, “coding-synonymous”, “stop-gained”, “missense-near-splice”, “coding-synonymous-near-splice”, “stop-lost”, “splice-5”, “splice-3”, “stop-gained-near-splice”, and “stop-lost-near-splice”, as provided by the Exome Sequencing Project, and described in Tennessen et al 2012. Of these variant annotations, we consider “missense”, “stop-gained”, “missense-near-splice”, “stop-lost”, “splice-5”, “splice-3”, “stop-gained-near-splice”, and “stop-lost-near-splice” as putatively “functional” variant annotations, while we considered “coding-synonymous” and “coding-synonymous-near-splice” as putatively “non-functional” variants.

Minor Allele Frequency: We rely on the combined EA and AA cohorts, and thus rely on the ESP6500 “All” component of column “MAFinPercent(EA/AA/All)” for the minor allele frequency of any given CCDS variant. For the primary analysis we consider the MAF cut-off at 0.1% frequency in the combined population. However, we further considered what effect on score alternating MAF cut-offs might have to better understand the residual variation intolerance scores' behaviour across frequency spectrum cut-offs of 0.01% and 1%, Figure S2.

### Investigating a variant-level informed Residual Variation Intolerance Score (RVIS-PP2)

To explore alternative genome-wide scoring that leverages from additional variant-level data we informed the RVIS score with the inclusion of PolyPhen-2 *in silico* predictions, as annotated in the NHLBI-ESP. We considered PolyPhen-2 “benign” qualitative assessments as “non-functional”, and PolyPhen-2 “probably, possibly, and unknown effects” as “functional”. Then, as before, we defined the threshold dividing “common” and “rare” as 0.1% minor allele frequency (MAF). We defined Y as the total number of common, MAF>ρ “functional” missense and “truncating” SNVs (including splice and nonsense) and let X be the total number of variants (including synonymous and “non-functional” missense mutations, regardless of frequency in the population) observed within a gene. We regressed Y on X and took the studentized residual as the score (S), as was described for the RVIS. In this manuscript, we refer to this revised RVIS score as the RVIS-PP2. The Pearson's *r* correlation comparing the RVIS and the RVIS-PP2 was 0.76 [95% CI 0.75–0.77]. Results of the correlation between the RVIS-PP2 and OMIM disease genes are presented in Table 1.

### Deriving the OMIM lists for score assessment

As a primary assessment of score behaviour, we determine how well the scores predict known gene-lists from six different contexts, extracted from the OMIM database (accessed 3^rd^ December 2012): OMIM disease genes, “recessive”, “haploinsufficiency”, “dominant-negative”, “*de novo*” disease-causing, and indirectly derive an OMIM “non-disease” gene list.

For the five disease gene lists we filter only for gene entries that are annotated with a (*) indicating genes with known sequence and (#) indicating that a phenotype description and molecular basis is known. Moreover, we restrict it to records with “Allelic variants” and a “Gene Map Locus”. For the “recessive” (n = 881 genes), “haploinsufficiency/haploinsufficient” (n = 251 genes), “dominant negative” (n = 387 genes) and “*de novo*” (n = 507 genes) lists, we adopted those keywords, understanding that pulling out by keyword will identify some instances where the keyword is used for one reason or another even though the gene in question does not follow the indicated genetic model. We directly estimate this misclassification rate by inspecting a random subset of 30 genes from each of the OMIM categories and found it varied from a zero misclassification rate to a maximum of 30%. For the “haploinsufficiency/haploinsufficient” list we did manually curate each event to restrict to events with a confident haploinsufficient relationship (n = 202 genes) (lists are available in Dataset S1).

For the OMIM disease gene list (n = 2,329) we did a universal capture of all genes linked to disease, excluding genes linked to disorders with the following criteria: “resistance”, “cancer”, “somatic”, “susceptibility”, “carcinoma” and “tumor”. We further refined that list to only genes without the following annotations: braces “{” reflecting mutations contributing to susceptibility to multifactorial or infectious diseases, brackets “[]” reflecting genes linked to non-disease traits and question mark “?” indicating an unconfirmed or possibly spurious mapping. We found that 56.5% of the genes from the OMIM disease gene list overlap with at least one of the four additional OMIM contexts, described earlier. Moreover, we observe that 5.3% of OMIM recessive genes were also annotated to OMIM haploinsufficiency, while 61.7% of OMIM haploinsufficiency genes overlapped with the “*de novo*” gene list (Dataset S1).

The OMIM non-disease gene list (n = 14,712 genes) is derived by excluding, from the list of 16,956 HGNC assessable genes, any genes overlapping with at least one of the five described OMIM disease gene lists.

### Comparison to omega, a measure of evolutionary selective pressure: Ka/Ks ratio

To compare the RVIS to measures of omega (*ω*), we consider HGNC genes in the subset of the human genome (chromosomes 1–5) that have been derived and kindly provided by Dr. Chuanzhu Fan [25]. Dr. Chuanzhu Fan and colleagues calculated *Ka/Ks* for the orthologs between human and chimps for chromosomes 1–5, using codeml [22], NG [24], and LWL [23]. For our comparisons, we relied on the subset of 2,963 genes across chromosomes 1–5, where a score was available for all four scoring systems: RVIS, codeml, LWL, and NG. Where a gene had multiple transcripts, we considered the average *Ka/Ks* across those transcripts for each omega scoring system. Across these 2,963 genes, the highest correlation between the four scores was found for the pair-wise comparison between LWW and NG (Pearson's *r* = 0.82), and the second highest was a Pearson's *r* of 0.11 for RVIS and codeml. Thus, it is clear that there is low correlation between the RVIS score and these ratios of *Ka/Ks*.

To address the question as to whether the *Ka/Ks* scores were better correlated to OMIM disease gene lists, we directly compared all four scores to the subset of gene annotations for the 2,963 genes. We found that, across the OMIM disease gene lists, the AUC consistently remained higher for the RVIS (Table S3). Most notably the *de novo* specific haploinsufficiency list, using RVIS as the predictor, obtained an AUC of 0.76 [95% CI 0.66–0.87], while, in comparison, the highest AUC among the three omega scores was for NG, AUC of 0.61 [95% CI 0.47–0.75]. The closest comparison between the RVIS score and the omega scores was for the All OMIM gene list, where the RVIS score obtained an AUC of 0.56 [95% CI 0.53–0.59], compared to NG, AUC = 0.52 [95% CI 0.49–0.55]. ROC curves for each of the investigated lists are available (Figure S4).

### Score sensitivity to sub-population and allele frequency

We assessed the sensitivity of the 0.1% Minor Allele Frequency (MAF) residual variation intolerance score in the combined ESP6500 population by comparing it to the European and African American subpopulations, and to varied thresholds of 0.01% and 1% MAF (Figure S2).

First, we regenerated the scores based on altering the MAF cut-off in the combined cohort from ρ = 0.1% to ρ = 0.01% and subsequently, ρ = 1%. We then compared the residual variation intolerance scores under the alternative MAF thresholds to that obtained using the 0.1% MAF. We obtained Pearson's *r* correlation coefficients of 0.849 [95%CI 0.845–0.853] comparing 0.1% MAF and 0.01% MAF, and 0.813 [95%CI 0.808–0.818] for the comparison between 0.1% MAF and 1% MAF (Figure S2 [A and B]).

We then regenerated the residual variation intolerance scores for the 0.1% MAF threshold based on the two sub-populations comprising the European Americans (EA) and African Americans (AA). In doing so, 124 (0.7%) of the 16956 HGNC assessable genes were identified as un-assessable for having insufficient coverage in one of the two separate populations, and were omitted from these comparisons. We found that the combined residual variation intolerance score (ALL) obtained a Pearson's *r* correlation coefficient of 0.862 [95%CI 0.858–0.865] for the comparison with the EA, and 0.908 [95%CI 0.905–0.911] for the comparison between AA and the combined (ALL) cohort (Figure S2 [C and D]).

We then investigated the effects on the MAF comparison when stratifying by sub-population to eliminate the effect of sample size differences in the MAF comparisons previously performed on the combined cohort of EA and AA. Using a MAF comparison of 0.1% and 1% in each of the EA and AA sub-populations, we obtain a Pearson's *r* correlation coefficient of 0.836 [0.832–0.841] for the EA 1% versus EA 0.1% MAF thresholds, and 0.850 [0.846–0.855] for the AA 1% versus AA 0.1% MAF thresholds (Figure S2 [E and F]). We could not do a similar comparison for the 0.1% versus 0.01% MAF threshold in the sub-populations due to resolution limitations at such a low frequency, but given the current evidence from the comparisons we are encouraged that it will remain high.

Finally, we showed that, while there is minor fluctuation in the curves, the signals did not differ when stratifying to the EA or AA sub-populations for the capacity to associate with OMIM disease genes (Figure S5). Likewise, the overall signals did not differ when adjusting ρ to 0.01% or 1.0% MAF for the capacity to associate with OMIM disease genes (Figure S6). The slight dip in performance for the 0.01% MAF is likely a result of the reduced resolution to sufficiently assess variants at that frequency level among a cohort of approximate 6503 combined samples.

We found no correlation, Pearson's *r* of 0.005 [95%CI −0.010–0.020], between the RVIS (0.1% MAF, combined population) to (X) the number of variants observed in the corresponding gene. This is consistent with the expectation that the raw residuals and X are independent by construction. Furthermore, there was a very weak correlation, Pearson's *r* correlation of −0.099 [95%CI −0.114–−0.084], between the RVIS and the coverage-corrected gene size. We did not find strong correlation between RVIS and the percentage GC content of the gene (www.ensembl.org/biomart/martview), Pearson's *r* of −0.03. Thus, it is clear that the information captured by the RVIS is not systematically biased by the number of variants in a gene, gene size, or the percentage GC content of the gene.

### Assessing additional gene lists

In addition to the primary OMIM gene lists, we assessed the behaviour of the residual variation intolerance score within four alternatively derived lists of interest. Two lists were derived from the Mouse Genome Informatics (MGI) database (last accessed 3^rd^ December 2012, http://www.informatics.jax.org/), and a third was the combination of overlapping entries between OMIM “haploinsufficient” and OMIM “*de novo*” lists (n = 108). The first MGI-derived list focused on “lethality” genes (n = 91), which represent human orthologs, with public CCDS transcript(s), where mouse knockouts have resulted in embryonic [MP:0008762], prenatal, [MP:0002080] or perinatal [MP:0002081] lethality. The second list focused on “seizure” genes (n = 95), which represent human orthologs, with public CCDS transcript(s), where mouse knockouts have resulted in a phenotype with a seizure presentation (MP:0002064). Gene lists are available in Dataset S1. While we do not expect all the mouse knockout “lethality” and “seizure” genes to have identical consequence in humans, they are comparable proxies that are expected to be enriched for genes that when disrupted could have comparable phenotypes.

A fourth list comprised of genes considered “essential” in a recent paper by Georgi et al. (2013) [21]. Of the 2,472 “essential” genes, 2,288 (92.6%) had an available RVIS score. The remaining 7.4% of “essential” genes were unavailable due to having either less than 70% of the gene assessed within the NHLBI-ESP, as described in earlier methods, or not matching a public CCDS Release 9 transcript.

### Assessing the disorder classes from the human disease network

To determine the disorder classes that are most likely to be affected by mutations in intolerant genes, we rely on previously curated lists of OMIM genes categorised into the 22 disorder classes by Goh et al. 2007 as part of the human disease network diseasome mapping effort [26]. The disorder class annotations are published in Goh et al. (2007) “Supporting Information Table 1”. [http://www.pnas.org/content/suppl/2007/05/03/0701361104.DC1/01361Table1.pdf - last accessed 27^th^ December 2012]. We filtered only for HGNC genes within the source list that were assigned an RVIS value. We summarized the RVIS within each of the 22 disorder classes (Table S2).

To compare RVIS values in an early versus late-onset genetic disorder context, we took epileptic encephalopathy (EE) genes from OMIM to represent “early-onset”: *ARX* (EIEE1 – OMIM# 308350), *CDKL5* (EIEE2 – OMIM# 300672), *SLC25A22* (EIEE3 – OMIM# 609304), *STXBP1* (EIEE4 – OMIM# 612164), *SPTAN1* (EIEE5 – OMIM# 613477), *SCN1A* (EIEE6 – OMIM# 607208), *KCNQ2* (EIEE7 – OMIM# 613720), *ARHGEF9* (EIEE8 – OMIM# 300607), *PCDH19* (EIEE9 – OMIM# 300088), *PNKP* (EIEE10 – OMIM# 613402), *SCN2A* (EIEE11 – OMIM# 613721), *PLCB1* (EIEE12 – OMIM# 613722), *SCN8A* (EIEE13 – OMIM# 614558), *KCNT1* (EIEE14 – OMIM# 614959), *MAPK10* (LGS EE – OMIM# 606369). Of these 16 EE genes, *ARX* was not assigned an RVIS score because it was insufficiently covered (less than 70% of gene) in the NHLBI-ESP (Methods). Of the remaining 15 genes, *ST3GAL3*, *ARHGEF9*, *SLC25A22*, *PNKP*, and *PLCB1* lacked OMIM annotation for a dominant model. The genes considered for amyotrophic lateral sclerosis (ALS), a “late-onset” severe neuronal disorder, were similarly extracted from OMIM: *SOD1* (ALS1 – OMIM# 105400), *ALS2* (ALS2 – OMIM# 205100), *SETX* (ALS4 – OMIM# 602433), *FUS* (ALS6 – OMIM# 608030), *VAPB* (ALS8 – OMIM# 608627), *ANG* (ALS9 – OMIM# 611895), *TARDBP* (ALS10 – OMIM# 612069), *FIG4* (ALS11 – OMIM# 612577), *OPTN* (ALS12 – OMIM# 613435), *VCP* (ALS14 – OMIM# 613954), *UBQLN2* (ALS15 – OMIM# 300857), *SIGMAR1* (ALS16 – OMIM# 614373), *CHMP2B* (ALS17 – OMIM# 614696), *PFN1* (ALS18 – OMIM# 614808), *C9orf72* (ALS – OMIM# 105550). Of the 15 ALS genes, *ALS2* and *SIGMAR1* lacked OMIM annotation for a dominant model. OMIM susceptibility genes “{” were not considered, and only genes with reported causal genetic variants were eligible.

### Assessing the trio sequencing studies across autism, severe ID, epileptic encephalopathies, and presumed non-neurologically impaired sibling controls

Using a 25^th^ percentile intolerance threshold to define the quarter of genes, genome-wide, that are most intolerant, we observed an increased enrichment of *de novo* mutations in the disease cohorts for the more damaging mutation types (Figure 5, Table S3). Larger numbers of sequenced trios among these groups will facilitate improved interpretation of the enrichment for *de novo* mutations in intolerant genes among children affected by neurological/developmental disorders. Limitations interpreting these data include that 6.1% of the *de novo* mutations reported from the autism studies arose from multiplex families. Moreover, there is literature supporting overlaps between autism, EE, and severe ID; however, the exact percentage of the autism samples sequenced across the four ASD studies that had severe ID, EE, or both, were not readily available.

### Utilizing a multidimensional mutation prioritization scheme

To illustrate constructing a multidimensional prioritizing scheme for mutations we first collect all the publically available *de novo* mutations published across the autism, severe ID, epileptic encephalopathies, and control data from recently published papers [9]–[15]. We collectively annotated all *de novo* mutations to extract the *de novo* missense mutations using ensembl variant effect predictor v2.6 (*Ve!P*). Only mutations reported in CCDS transcripts [17] were considered. Restricting to missense CCDS mutations, for each *de novo* mutation we consider the most damaging PolyPhen-2 CCDS annotation. As the most likely *de novo* mutation genetic model is a single causal *de novo* mutation, for samples with multiple missense *de novo* mutations, we used the single most damaging *de novo* based on the lowest RVIS value (i.e., the most intolerant gene affected). Finally, we split the remaining pooled *de novo* missense mutations into the four groups: Control (Figure 6A), Severe ID (Figure 6B), Epileptic Encephalopathy (Figure 6C), and Autism (Figure 6D).

We plotted each of the *de novo* missense mutations in the 2D space (x-axis = PolyPhen-2 quantitative score; y-axis = Residual Variation Intolerance Score percentile). We considered the high-interest region “hot zone” to correspond to highly-predicted “functionally damaging” PolyPhen-2 missense mutations (x≥0.95), and RVIS within the lowest 25% of genes (y≤0.25) (Figures 6 [A–D]). We list the *de novo* mutations within the high-interest region, for each cohort, in Dataset S3. While other elaborations of this multidimensional approach are possible, including higher dimensions that incorporate additional variant-level quantitative scores, such as SIFT, GERP++, MAPP, etc., here we aim to provide the simplest proof-of-concept for how this can be conceptualized, and ultimately adopted within relevant contexts.

For simplicity we presented only 2D plots that considered the missense *de novo* mutations from the corresponding studies (Figure 6). However, it is certainly plausible to incorporate the information from other SNV effect types. For example, nonsense and essential splice site SNVs can be included in the assessment under a recoded PolyPhen-2 probabilistic damaging score of 1, likewise, silent *de novo* mutations can be recoded with a probabilistic damaging score of 0. With the inclusion of these additional SNV effect types, the preferential enrichment for each of the cohorts in this most damaging “hot zone” (PolyPhen-2≥0.95 and RVIS≤0.25) for controls is 11.54%, compared to severe ID (48.96%, p = 9.4×10^−14^, 2-tail Fisher's Exact test); EE (30.86%, p = 5.9×10^−7^, 2-tail Fisher's Exact test); and ASD (23.25%, p = 1.9×10^−5^, 2-tail Fisher's Exact test).

## Supporting Information

Dataset S1 The adopted OMIM and MGI gene lists (last accessed 3^rd^ December 2012).
(XLSX)

Dataset S2 The Residual Variation Intolerance Score (RVIS) and corresponding RVIS percentile for the full set of 16,956 CCDS assessed genes.
(XLSX)

Dataset S3 Details of the missense *de novo* mutations that are located within the high-interest region (PolyPhen-2 > = 0.95 and RVIS Percentile < = 0.25) for each of the control, severe intellectual disability, epileptic encephalopathy, and autism spectrum disorder cohorts. Putative loss of function SNV *de novo* mutations among the intolerant genes are also provided, but not plotted in Figure 6.
(XLSX)

Figure S1 Histogram of the Residual Variation Intolerance Scores (RVIS), with annotations for each of the 25% boundaries. Red: ≤25^th^ percentile of genes, Orange: >25^th^ and ≤50^th^ percentile, Grey: >50^th^ and ≤75^th^ percentile, Blue: >75^th^ percentile.
(EPS)

Figure S2 Scatter plots reflecting correlations between RVIS derived from alternating population and minor allele frequency (MAF) thresholds. [A] Combined population RVIS: 0.1% MAF vs. 0.01% MAF; [B] Combined population RVIS: 0.1% MAF vs. 1% MAF; [C] 0.1% RVIS: Combined population vs. European Americans; [D] 0.1% RVIS: Combined population vs. African Americans; [E] European Americans RVIS: 0.1% MAF vs. 1% MAF; [F] African Americans RVIS: 0.1% MAF vs. 1% MAF; [G] 0.1% MAF RVIS: European Americans vs. African Americans; [H] Combined population RVIS: 0.01% MAF vs. 1% MAF.
(PDF)

Figure S3 Receiver Operating Characteristic (ROC) curves illustrating RVIS-PP2 in the context of the OMIM disease gene lists.
(EPS)

Figure S4 Receiver Operating Characteristic (ROC) curves comparing RVIS to estimates of omega *(Ka/Ks)* across OMIM and MGI disease gene lists for chromosomes 1–5.
(PDF)

Figure S5 Receiver Operating Characteristic (ROC) curves of the alternating population on the association between the RVIS and predicting OMIM disease data.
(TIF)

Figure S6 Receiver Operating Characteristic (ROC) curves of the alternating minor allele frequency (MAF) thresholds on the association between the RVIS and predicting OMIM disease data.
(TIF)

Table S1 Comparing the residual variation intolerance score (RVIS) to the three sources of omega estimates (*ω*). AUC estimates are based on lower scores being predictive of corresponding gene list.
(DOCX)

Table S2 Profiling the residual variation intolerance score among the 22 disorder classes defined by the human disease network [26].
(DOCX)

Table S3 Profiling the residual variation intolerance score (RVIS), across three *de novo* mutant functional-effect categories, among the published trio sequencing studies of severe ID, epileptic encephalopathies, autism, and sibling controls.
(DOCX)

## References

[1] Davydov EV, Goode DL, Sirota M, Cooper GM, Sidow A, et al (2010) Identifying a high fraction of the human genome to be under selective constraint using GERP++. PLoS Comput Biol 6: e1001025. 21152010 10.1371/journal.pcbi.1001025 PMC2996323

[2] Adzhubei IA, Schmidt S, Peshkin L, Ramensky VE, Gerasimova A, et al (2010) A method and server for predicting damaging missense mutations. Nat Methods 7: 248–249. 20354512 10.1038/nmeth0410-248 PMC2855889

[3] Lee W, Yue P, Zhang Z (2009) Analytical methods for inferring functional effects of single base pair substitutions in human cancers. Hum Genet 126: 481–498. 19434427 10.1007/s00439-009-0677-y PMC2762536

[4] Sim NL, Kumar P, Hu J, Henikoff S, Schneider G, et al (2012) SIFT web server: predicting effects of amino acid substitutions on proteins. Nucleic Acids Res 40: W452–457. 22689647 10.1093/nar/gks539 PMC3394338

[5] Hicks S, Wheeler DA, Plon SE, Kimmel M (2011) Prediction of missense mutation functionality depends on both the algorithm and sequence alignment employed. Hum Mutat 32: 661–668. 21480434 10.1002/humu.21490 PMC4154965

[6] Cooper GM, Shendure J (2011) Needles in stacks of needles: finding disease-causal variants in a wealth of genomic data. Nat Rev Genet 12: 628–640. 21850043 10.1038/nrg3046

[7] EVS. Exome Variant Server, NHLBI GO Exome Sequencing Project (ESP). Seattle, WA, accessed 3rd August 2012.

[8] OMIM (2012) Online Mendelian Inheritance in Man, OMIM. Baltimore, MD: McKusick-Nathans Institute of Genetic Medicine, John Hopkins University.

[9] Neale BM, Kou Y, Liu L, Ma'ayan A, Samocha KE, et al (2012) Patterns and rates of exonic de novo mutations in autism spectrum disorders. Nature 485: 242–245. 22495311 10.1038/nature11011 PMC3613847

[10] O'Roak BJ, Vives L, Girirajan S, Karakoc E, Krumm N, et al (2012) Sporadic autism exomes reveal a highly interconnected protein network of de novo mutations. Nature 485: 246–250. 22495309 10.1038/nature10989 PMC3350576

[11] Sanders SJ, Murtha MT, Gupta AR, Murdoch JD, Raubeson MJ, et al (2012) De novo mutations revealed by whole-exome sequencing are strongly associated with autism. Nature 485: 237–241. 22495306 10.1038/nature10945 PMC3667984

[12] de Ligt J, Willemsen MH, van Bon BW, Kleefstra T, Yntema HG, et al (2012) Diagnostic exome sequencing in persons with severe intellectual disability. N Engl J Med 367: 1921–1929. 23033978 10.1056/NEJMoa1206524

[13] Rauch A, Wieczorek D, Graf E, Wieland T, Endele S, et al (2012) Range of genetic mutations associated with severe non-syndromic sporadic intellectual disability: an exome sequencing study. Lancet 380: 1674–1682. 23020937 10.1016/S0140-6736(12)61480-9

[14] Iossifov I, Ronemus M, Levy D, Wang Z, Hakker I, et al (2012) De novo gene disruptions in children on the autistic spectrum. Neuron 74: 285–299. 22542183 10.1016/j.neuron.2012.04.009 PMC3619976

[15] Epi4K-Consortium (2013) De novo mutation in the classic epileptic encephalopathies. Nature [In Press].

[16] Tennessen JA, Bigham AW, O'Connor TD, Fu W, Kenny EE, et al (2012) Evolution and functional impact of rare coding variation from deep sequencing of human exomes. Science 337: 64–69. 22604720 10.1126/science.1219240 PMC3708544

[17] Pruitt KD, Harrow J, Harte RA, Wallin C, Diekhans M, et al (2009) The consensus coding sequence (CCDS) project: Identifying a common protein-coding gene set for the human and mouse genomes. Genome Res 19: 1316–1323. 19498102 10.1101/gr.080531.108 PMC2704439

[18] McKenna A, Hanna M, Banks E, Sivachenko A, Cibulskis K, et al (2010) The Genome Analysis Toolkit: a MapReduce framework for analyzing next-generation DNA sequencing data. Genome Res 20: 1297–1303. 20644199 10.1101/gr.107524.110 PMC2928508

[19] Heinzen EL, Swoboda KJ, Hitomi Y, Gurrieri F, Nicole S, et al (2012) De novo mutations in ATP1A3 cause alternating hemiplegia of childhood. Nat Genet 44: 1030–1034. 22842232 10.1038/ng.2358 PMC3442240

[20] Eppig JT, Blake JA, Bult CJ, Kadin JA, Richardson JE, et al (2012) The Mouse Genome Database (MGD): comprehensive resource for genetics and genomics of the laboratory mouse. Nucleic Acids Res 40: D881–886. 22075990 10.1093/nar/gkr974 PMC3245042

[21] Georgi B, Voight BF, Bucan M (2013) From mouse to human: evolutionary genomics analysis of human orthologs of essential genes. PLoS Genet 9: e1003484. 23675308 10.1371/journal.pgen.1003484 PMC3649967

[22] Goldman N, Yang Z (1994) A codon-based model of nucleotide substitution for protein-coding DNA sequences. Mol Biol Evol 11: 725–736. 7968486 10.1093/oxfordjournals.molbev.a040153

[23] Li WH, Wu CI, Luo CC (1985) A new method for estimating synonymous and nonsynonymous rates of nucleotide substitution considering the relative likelihood of nucleotide and codon changes. Mol Biol Evol 2: 150–174. 3916709 10.1093/oxfordjournals.molbev.a040343

[24] Nei M, Gojobori T (1986) Simple methods for estimating the numbers of synonymous and nonsynonymous nucleotide substitutions. Mol Biol Evol 3: 418–426. 3444411 10.1093/oxfordjournals.molbev.a040410

[25] Zhang C, Wang J, Long M, Fan C (2013) gKaKs: the pipeline for genome-level Ka/Ks calculation. Bioinformatics 29: 645–646. 23314322 10.1093/bioinformatics/btt009

[26] Goh KI, Cusick ME, Valle D, Childs B, Vidal M, et al (2007) The human disease network. Proc Natl Acad Sci U S A 104: 8685–8690. 17502601 10.1073/pnas.0701361104 PMC1885563

[27] DeLong ER, DeLong DM, Clarke-Pearson DL (1988) Comparing the areas under two or more correlated receiver operating characteristic curves: a nonparametric approach. Biometrics 44: 837–845. 3203132

[28] Robin X, Turck N, Hainard A, Tiberti N, Lisacek F, et al (2011) pROC: an open-source package for R and S+ to analyze and compare ROC curves. BMC Bioinformatics 12: 77. 21414208 10.1186/1471-2105-12-77 PMC3068975

