# Correction: Genic Intolerance to Functional Variation and the Interpretation of Personal Genomes

**DOI:** 10.1371/annotation/32c8d343-9e1d-46c6-bfd4-b0cd3fb7a97e

**Published:** 2013-08-23

**Authors:** Slavé Petrovski, Quanli Wang, Erin L. Heinzen, Andrew S. Allen, David B. Goldstein

The following information was missing from the Funding section: 

This work was supported in part by the NIH Epi4K Sequencing, Bioinformatics and Biostatistics Core grant number U01NS077303. 

There was an error in reference 15. The correct reference is: 

Epi4K Consortium & Epilepsy Phenome/Genome Project (2013). De novo mutations in epileptic encephalopathies. *Nature*. Advance online publication. doi:10.1038/nature12439 

