# Supplementary material for: Genic Intolerance to Functional Variation and the Interpretation of Personal Genomes
Source: PLoS Genet. 2013 Aug 22;9(8):e1003709. doi: 10.1371/journal.pgen.1003709 (PMC3749936; doi:10.1371/journal.pgen.1003709)

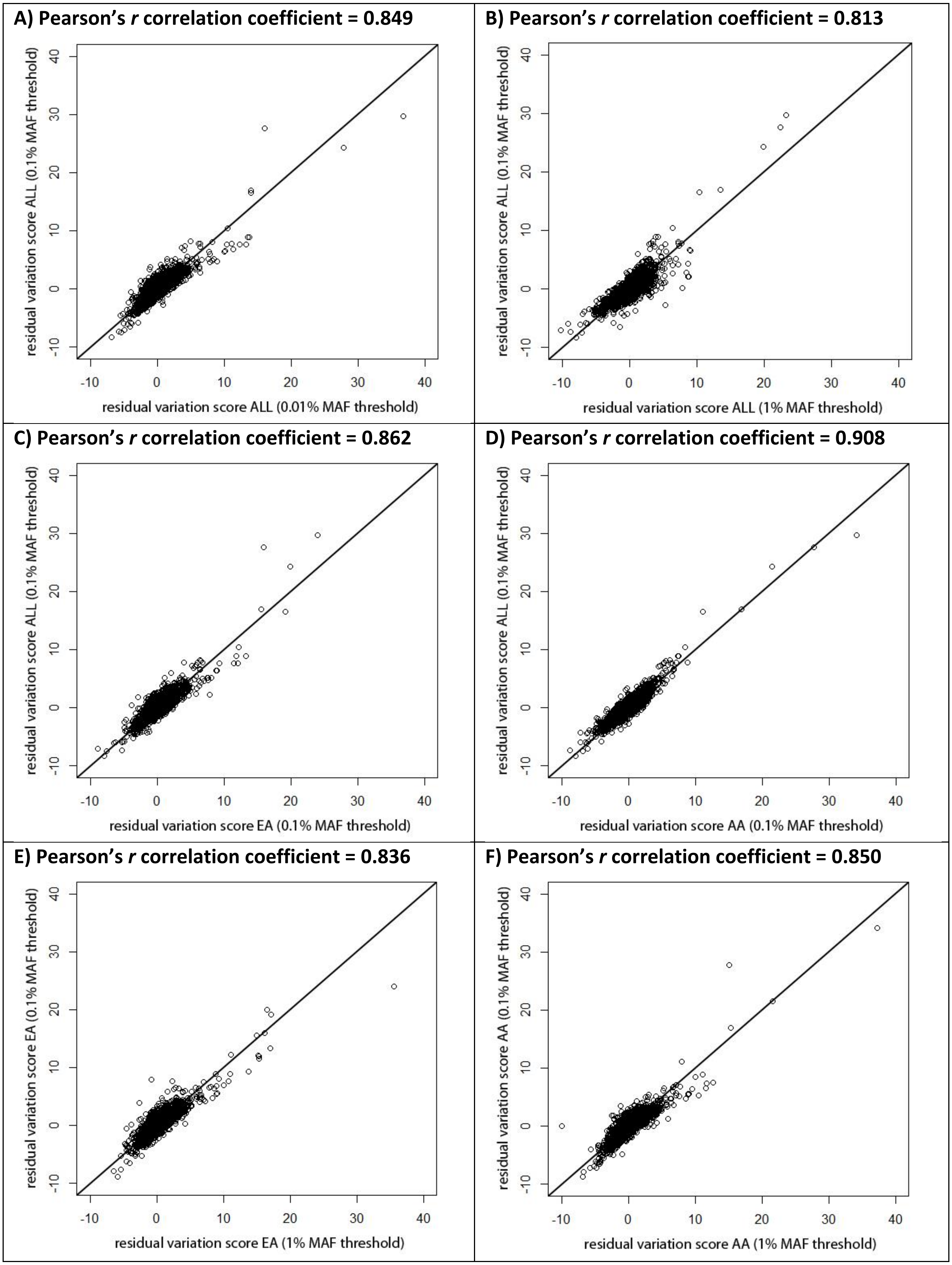
A) Pearson's r correlation coefficient = 0.849
residual variation score ALL (0.1% MAF threshold)
residual variation score ALL (0.01% MAF threshold)
B) Pearson's r correlation coefficient = 0.813
residual variation score ALL (0.1% MAF threshold)
residual variation score ALL (1% MAF threshold)
C) Pearson's r correlation coefficient = 0.862
residual variation score ALL (0.1% MAF threshold)
residual variation score EA (0.1% MAF threshold)
D) Pearson's r correlation coefficient = 0.908
residual variation score ALL (0.1% MAF threshold)
residual variation score AA (0.1% MAF threshold)
E) Pearson's r correlation coefficient = 0.836
residual variation score EA (0.1% MAF threshold)
residual variation score EA (1% MAF threshold)
F) Pearson's r correlation coefficient = 0.850
residual variation score AA (0.1% MAF threshold)
residual variation score AA (1% MAF threshold)
-10
0
10
20
30
40

G) Pearson's *r* correlation coefficient = 0.733

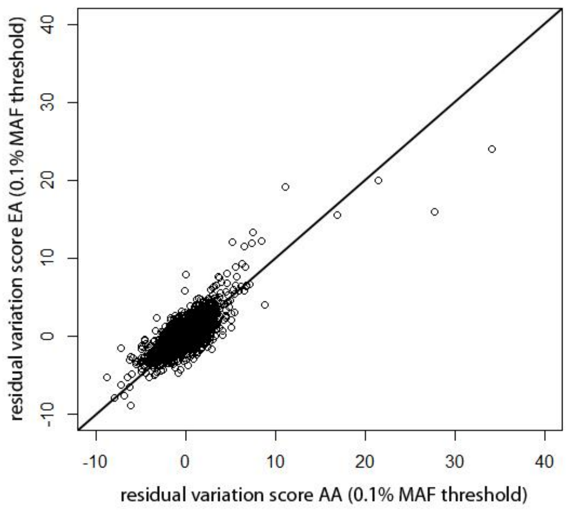

H) Pearson's *r* correlation coefficient = 0.685

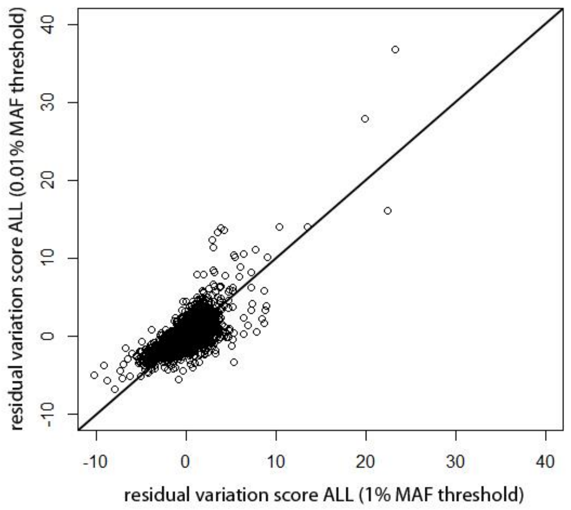

Supplement: Figure S2 — Scatter plots reflecting correlations between RVIS derived from alternating population and minor allele frequency (MAF) thresholds. [A] Combined population RVIS: 0.1% MAF vs. 0.01% MAF; [B] Combined population RVIS: 0.1% MAF vs. 1% MAF; [C] 0.1% RVIS: Combined population vs. European Americans; [D] 0.1% RVIS: Combined population vs. African Americans; [E] European Americans RVIS: 0.1% MAF vs. 1% MAF; [F] African Americans RVIS: 0.1% MAF vs. 1% MAF; [G] 0.1% MAF RVIS: European Americans vs. African Americans; [H] Combined population RVIS: 0.01% MAF vs. 1% MAF. (PDF) [file pgen.1003709.s005.pdf]
