# Supplementary figures and images for: Genic Intolerance to Functional Variation and the Interpretation of Personal Genomes
Source: PLoS Genet. 2013 Aug 22;9(8):e1003709. doi: 10.1371/journal.pgen.1003709 (PMC3749936; doi:10.1371/journal.pgen.1003709)

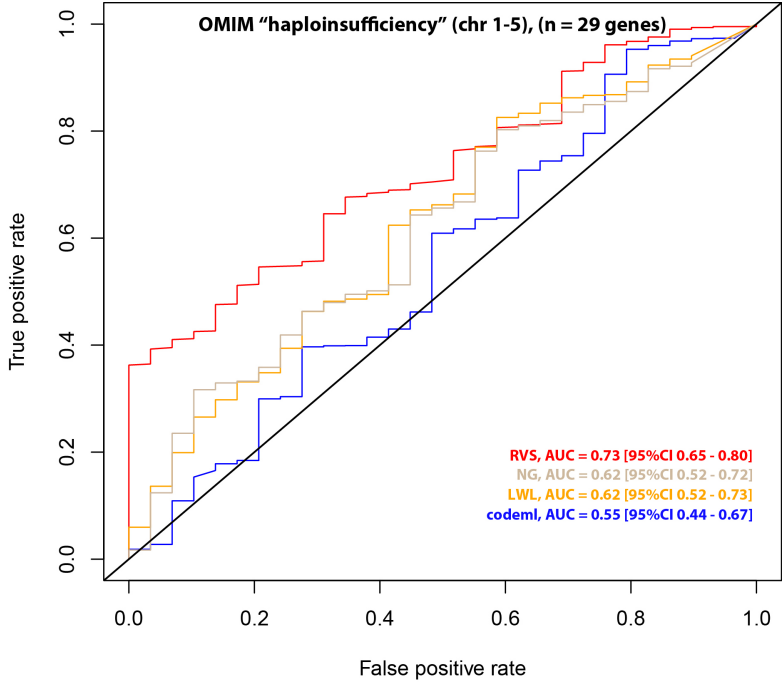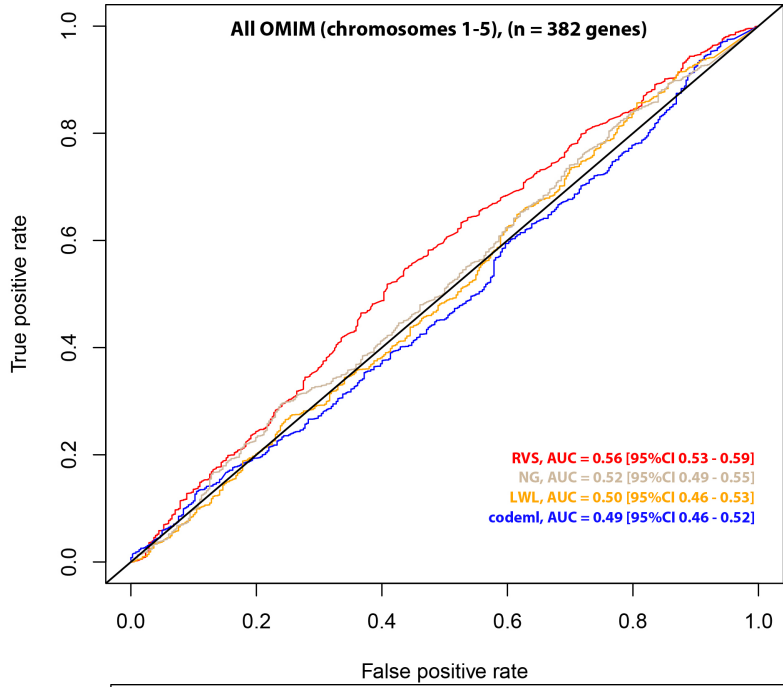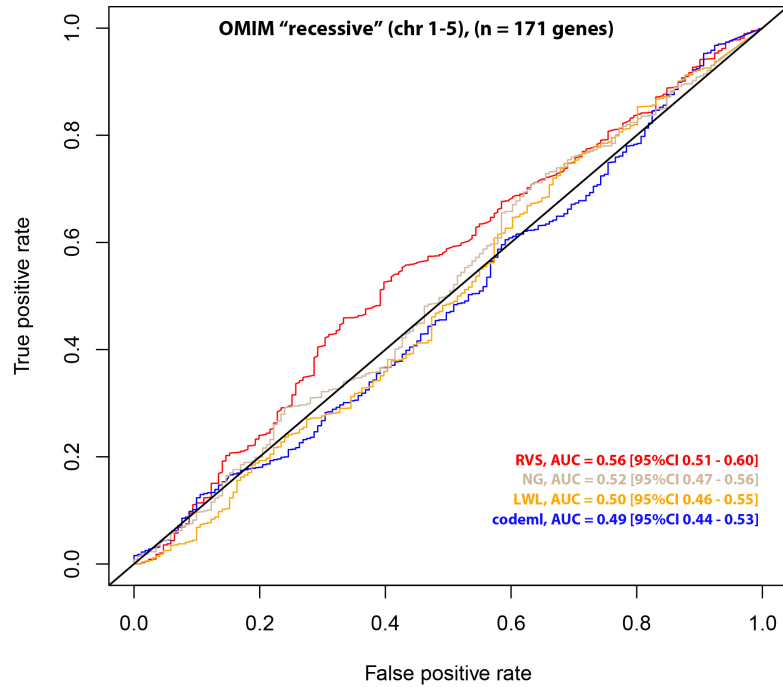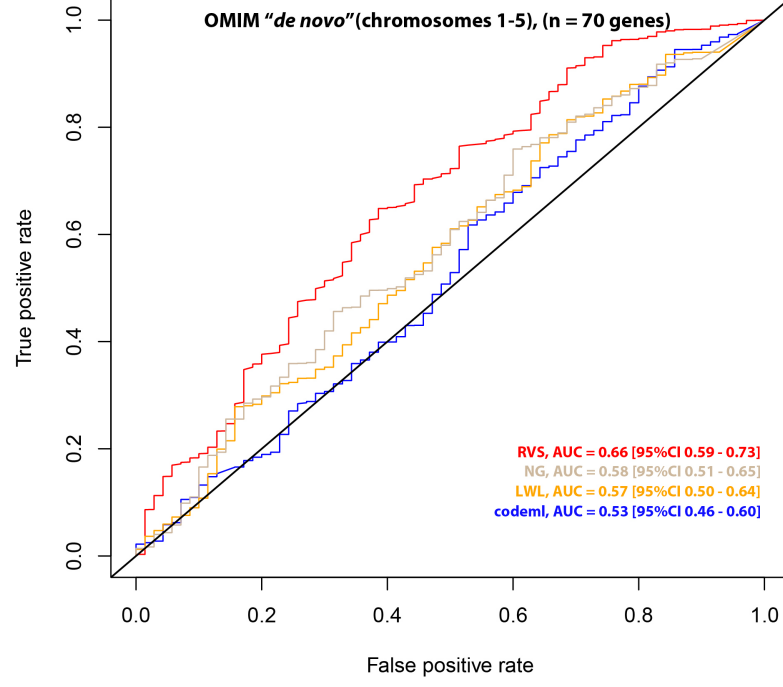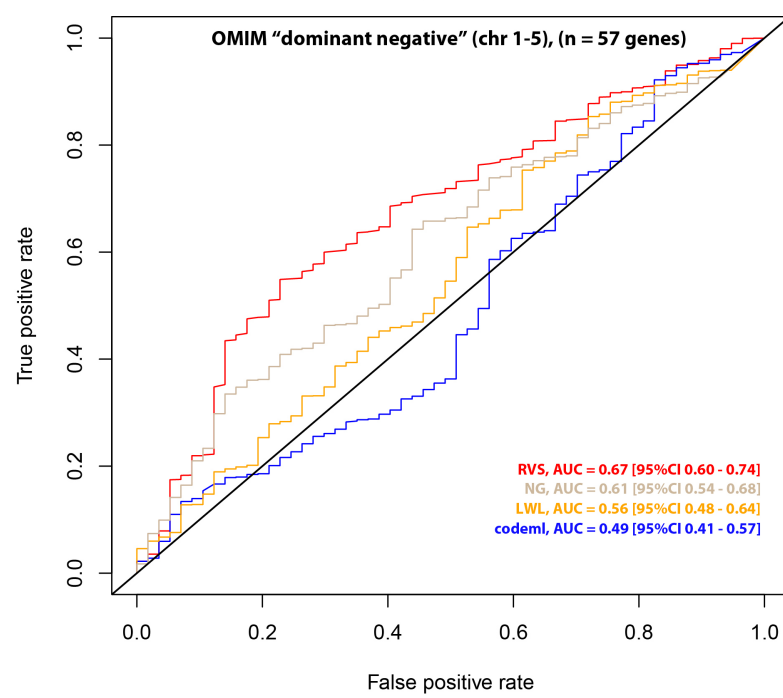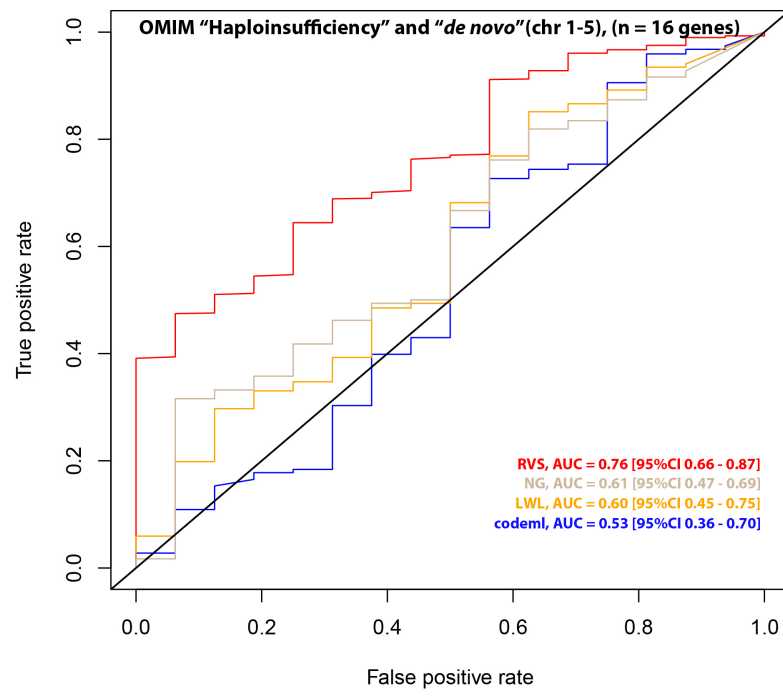

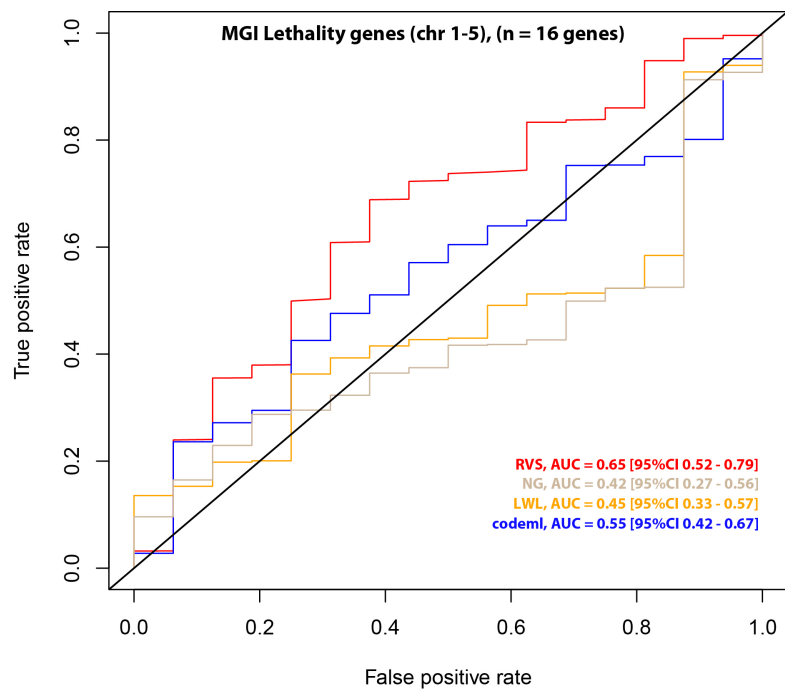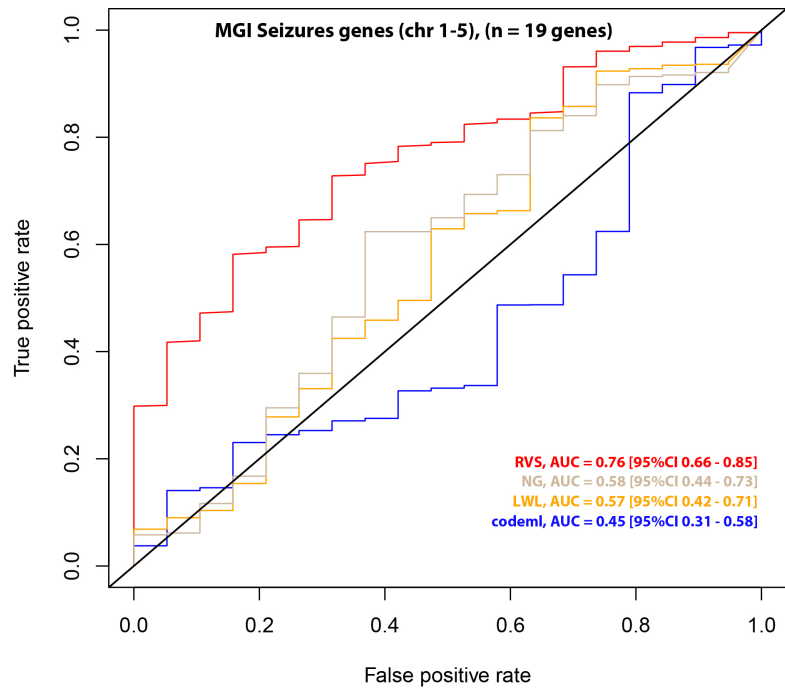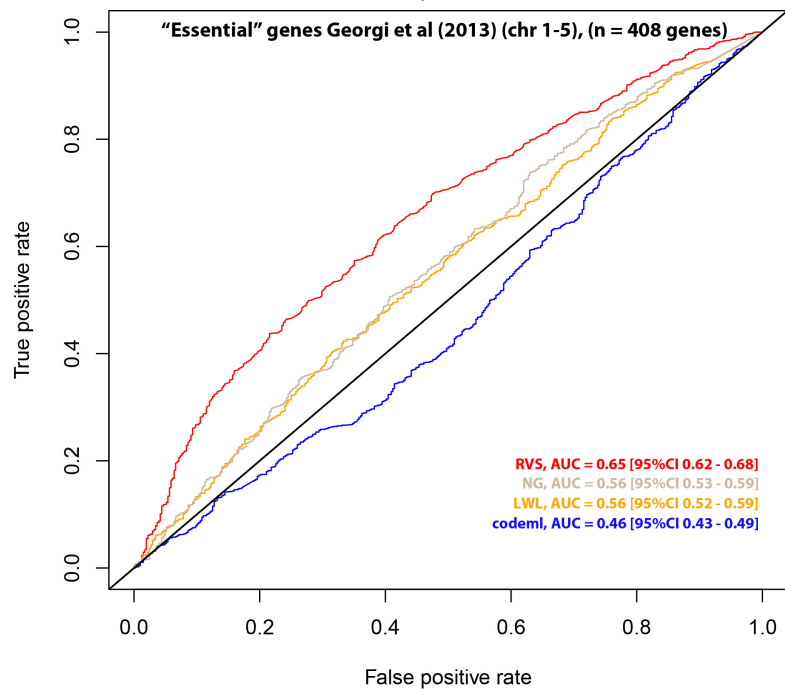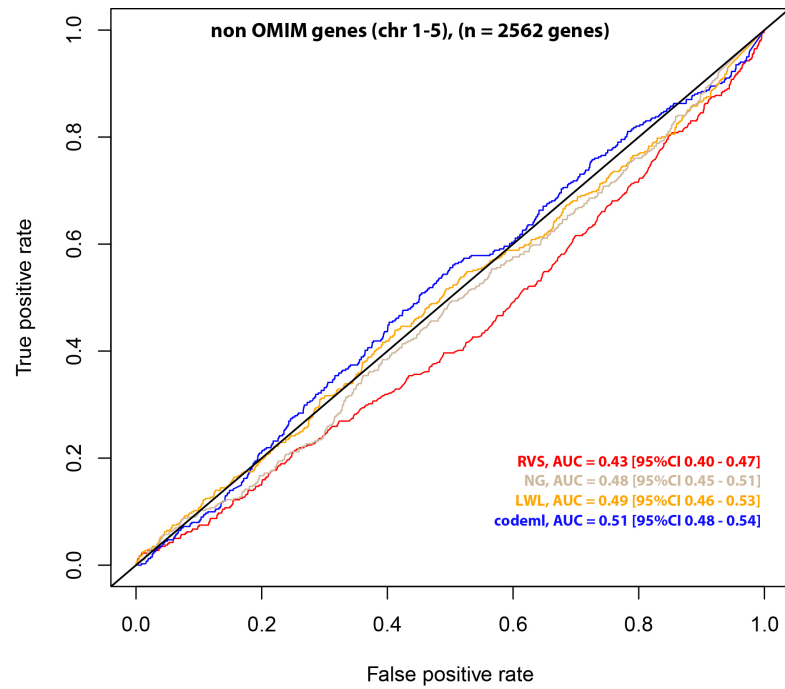

Supplement: Figure S4 — Receiver Operating Characteristic (ROC) curves comparing RVIS to estimates of omega (Ka/Ks) across OMIM and MGI disease gene lists for chromosomes 1–5. (PDF) [file pgen.1003709.s007.pdf]

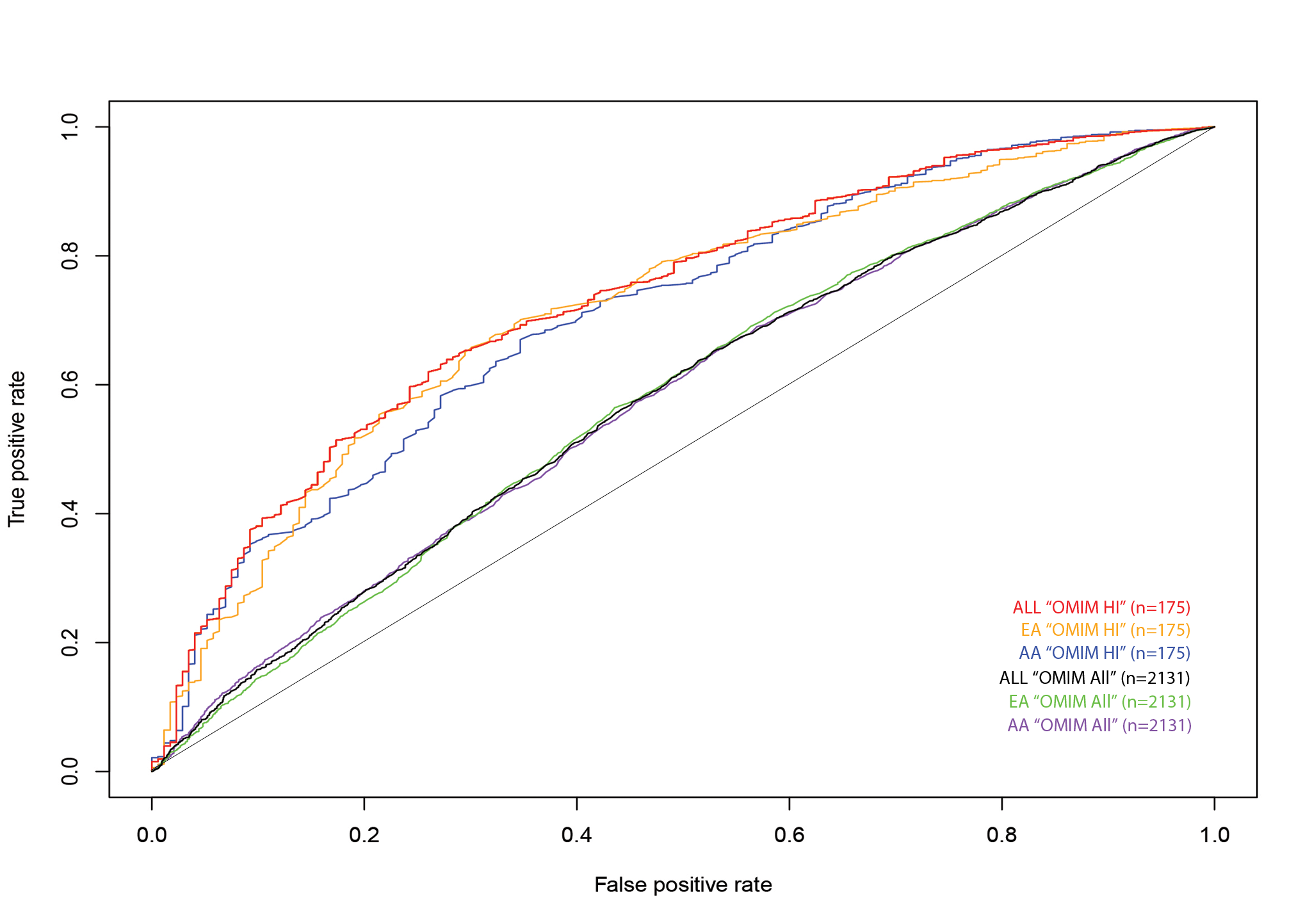

Supplement: Figure S5 — Receiver Operating Characteristic (ROC) curves of the alternating population on the association between the RVIS and predicting OMIM disease data. (TIF) [file pgen.1003709.s008.tif]

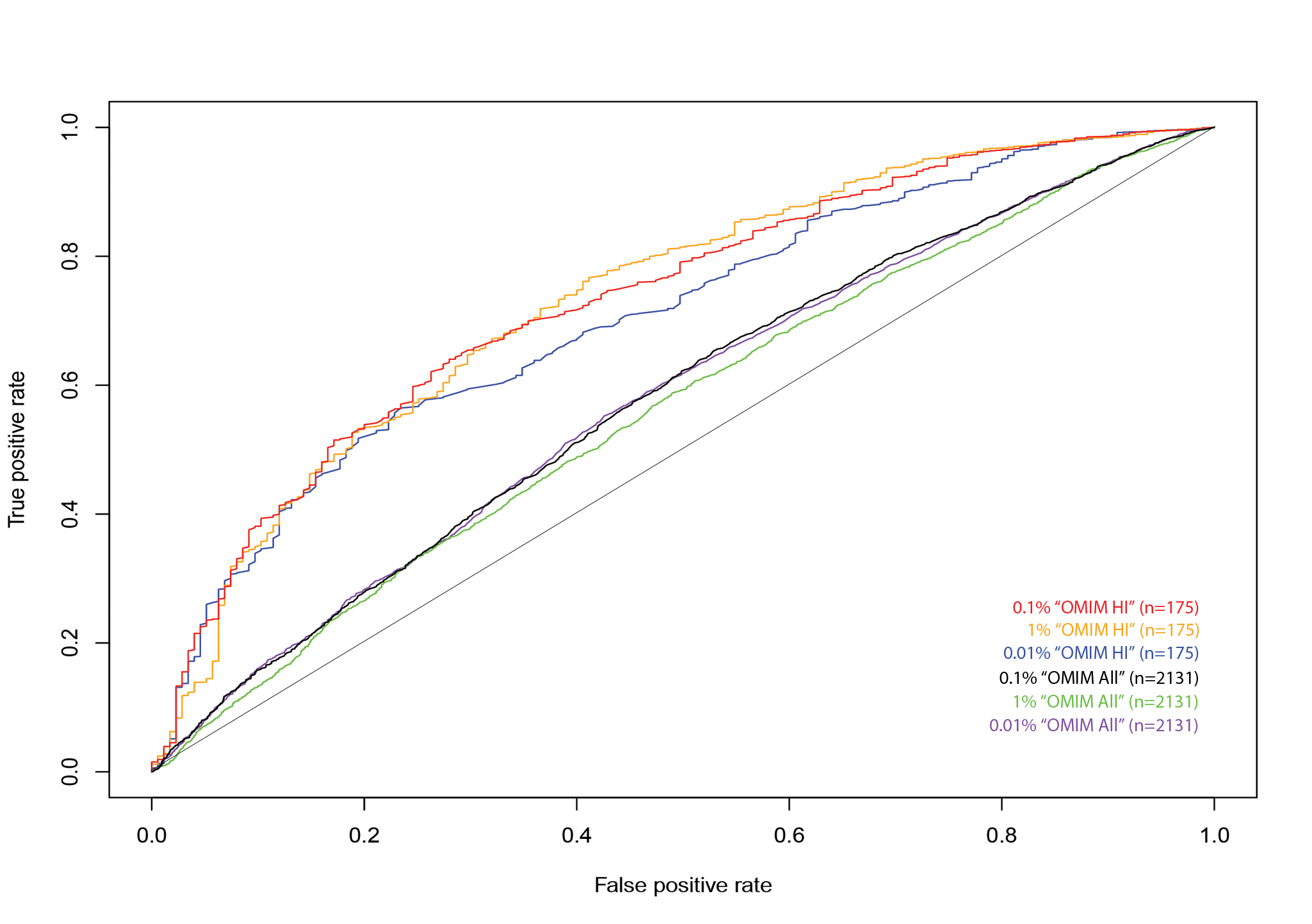

Supplement: Figure S6 — Receiver Operating Characteristic (ROC) curves of the alternating minor allele frequency (MAF) thresholds on the association between the RVIS and predicting OMIM disease data. (TIF) [file pgen.1003709.s009.tif]
