# Supplementary material for: Genic Intolerance to Functional Variation and the Interpretation of Personal Genomes
Source: PLoS Genet. 2013 Aug 22;9(8):e1003709. doi: 10.1371/journal.pgen.1003709 (PMC3749936; doi:10.1371/journal.pgen.1003709)
Supplement: Table S1 — Comparing the residual variation intolerance score (RVIS) to the three sources of omega estimates (ω). AUC estimates are based on lower scores being predictive of corresponding gene list. (DOCX) [file pgen.1003709.s010.docx]

**Table S1:** Comparing the residual variation intolerance score (RVIS) to the three sources of omega estimates (*ω*). AUC estimates are based on lower scores being predictive of corresponding gene list.

| **Gene List*** | **codeml [**[**22**](#_ENREF_22)**]** | **LWL [**[**23**](#_ENREF_23)**]** | **NG [**[**24**](#_ENREF_24)**]** | **RVIS** |
| --- | --- | --- | --- | --- |
| “Haploinsufficiency” (n=29) | **0.55**  [95% CI 0.44 – 0.67] | **0.62**  [95% CI 0.52 – 0.73] | **0.62**  [95% CI 0.52 – 0.72] | **0.73**  [95% CI 0.65 – 0.80] |
| OMIM disease genes (n=382) | **0.49**  [95% CI 0.46 – 0.52] | **0.5**  [95% CI 0.46 – 0.53] | **0.52**  [95% CI 0.49 – 0.55] | **0.56**  [95% CI 0.53 – 0.59] |
| “recessive” (n=171) | **0.49**  [95% CI 0.44 – 0.53] | **0.5**  [95% CI 0.46 – 0.55] | **0.52**  [95% CI 0.47 – 0.56] | **0.56**  [95% CI 0.51 – 0.60] |
| “*de novo*” (n=70) | **0.53**  [95% CI 0.46 – 0.60] | **0.57**  [95% CI 0.50 – 0.64] | **0.58**  [95% CI 0.51 – 0.65] | **0.66**  [95% CI 0.59 – 0.73] |
| “dominant negative” (n=57) | **0.49**  [95% CI 0.41 – 0.57] | **0.56**  [95% CI 0.48 – 0.64] | **0.61**  [95% CI 0.54 – 0.68] | **0.67**  [95% CI 0.60 – 0.74] |
| “Haploinsufficiency” and “*de novo*” (n=16) | **0.53**  [95% CI 0.36 – 0.70] | **0.6**  [95% CI 0.45 – 0.75] | **0.61**  [95% CI 0.47 – 0.69] | **0.76**  [95% CI 0.66 – 0.87] |
| MGI ortholog “lethality” (n=16) | **0.55**  [95% CI 0.42 – 0.67] | **0.45**  [95% CI 0.33 – 0.57] | **0.42**  [95% CI 0.27 – 0.56] | **0.65**  [95% CI 0.52 – 0.79] |
| MGI ortholog “seizures” (n=19) | **0.45**  [95% CI 0.31 – 0.58] | **0.57**  [95% CI 0.42 – 0.71] | **0.58**  [95% CI 0.44 – 0.73] | **0.76**  [95% CI 0.66 – 0.85] |
| Essential Gene List^ (n=408) | **0.46**  [95% CI 0.43 – 0.49] | **0.56**  [95% CI 0.52 – 0.59] | **0.56**  [95% CI 0.53 – 0.59] | **0.65**  [95% CI 0.62 – 0.68] |
| non OMIM Gene List (n=2562) | **0.51**  [95% CI 0.48 – 0.54] | **0.49**  [95% CI 0.46 – 0.53] | **0.48**  [95% CI 0.45 – 0.51] | **0.43**  [95% CI 0.40 – 0.47] |

*Gene lists were truncated to the 2,963 genes overlapping with the chromosome 1 – 5 HGNC genes.
*^ Essential gene list was extracted from Georgi et al (2013). [*[*21*](#_ENREF_21)*] Described in Method*
