# Supplementary material for: Genic Intolerance to Functional Variation and the Interpretation of Personal Genomes
Source: PLoS Genet. 2013 Aug 22;9(8):e1003709. doi: 10.1371/journal.pgen.1003709 (PMC3749936; doi:10.1371/journal.pgen.1003709)
Supplement: Table S2 — Profiling the residual variation intolerance score among the 22 disorder classes defined by the human disease network [26]. (DOCX) [file pgen.1003709.s011.docx]

**Table S2:** Profiling the residual variation intolerance score amongst the 22 disorder classes defined by the human disease network [[26](#_ENREF_26)].

| Disorder Class | Source list^1^ | Post filtering [%]^2^ | Average RVIS | 2-tail Mann-Whittney U test^3^  [sample estimate: difference in medians] |
| --- | --- | --- | --- | --- |
| Bone | 44 | 44 [100%] | -0.17 | 0.18 [-0.15] |
| Cancer* | 209 | 202 [96.7%] | -0.30 | 9.9x10^-9^ [-0.27]* |
| Cardiovascular* | 96 | 89 [92.7%] | -0.45 | 6.5x10^-6^ [-0.33]* |
| Connective tissue disorder | 52 | 47 [90.4%] | -0.24 | 5.5x10^-3^ [-0.31] |
| Dermatological | 83 | 75 [90.4%] | -0.07 | 0.89 [-0.01] |
| Developmental* | 53 | 46 [86.8%] | **-0.56** | 6.2x10^-5^ **[-0.45]*** |
| Ear, Nose, and Throat | 44 | 42 [95.5%] | -0.40 | 3.8x10^-3^ [-0.38] |
| Endocrine | 96 | 82 [85.4%] | -0.20 | 0.02 [-0.16] |
| Gastrointestinal | 34 | 32 [94.1%] | -0.09 | 0.47 [-0.09] |
| Hematological | 149 | 133 [89.3%] | 0.06 | 0.98 [-0.002] |
| Immunological | 119 | 108 [90.8%] | **0.26** | 0.03 **[0.14]** |
| Metabolic | 291 | 283 [97.3%] | -0.08 | 9.5x10^-3^ [-0.10] |
| Multiple* | 212 | 195 [92.0%] | -0.33 | 6.2x10^-9^ [-0.32]* |
| Muscular* | 70 | 64 [94.3%] | -0.52 | 9.0x10^-4^ [-0.30]* |
| Neurological* | 258 | 245 [95.0%] | -0.26 | 2.4x10^-8^ [-0.25]* |
| Nutritional | 23 | 21 [91.3%] | -0.05 | 0.81 [-0.03] |
| Ophthamological | 120 | 103 [85.8%] | -0.20 | 0.06 [-0.13] |
| Psychiatric | 30 | 27 [90.0%] | -0.08 | 0.45 [-0.11] |
| Renal | 59 | 55 [93.2%] | -0.19 | 0.03 [-0.21] |
| Respiratory | 34 | 34 [100%] | -0.08 | 1.00 [4x10^-5^] |
| Skeletal* | 58 | 48 [82.8%] | -0.36 | 2.4x10^-4^ [-0.37]* |
| Unclassified | 30 | 27 [90.0%] | -0.01 | 0.86 [0.02] |

*^1^Source list – reflects the number of unique genes within a disorder class (each gene considered once within a class, but can be present across multiple classes).*

*^2^Post filtering – reflects the number of unique genes remaining after excluding non-CCDS genes and genes that were un-assessable in the ESP6500 database (Methods).*

*^3^Mann-Whitney U test compares gene list of disorder class to the 14,712 genes in the non OMIM gene list (Supplementary Appendix 1).*

**significant for the number of tests (n=22), adjusted 0.05 alpha of 2.3x10^-3^.*
