# Supplementary material for: Genic Intolerance to Functional Variation and the Interpretation of Personal Genomes
Source: PLoS Genet. 2013 Aug 22;9(8):e1003709. doi: 10.1371/journal.pgen.1003709 (PMC3749936; doi:10.1371/journal.pgen.1003709)
Supplement: Table S3 — Profiling the residual variation intolerance score (RVIS), across three de novo mutant functional-effect categories, among the published trio sequencing studies of severe ID, epileptic encephalopathies, autism, and sibling controls. (DOCX) [file pgen.1003709.s012.docx]

**Table S3:** Profiling the residual variation intolerance score (RVIS), across three *de novo* mutant functional-effect categories, amongst the published trio sequencing studies of severe ID, epileptic encephalopathies, autism, and sibling controls.

|  | SILENT | | MISSENSE | | LGD | |
| --- | --- | --- | --- | --- | --- | --- |
| STUDY*^ | scored *de novo* / all *de novos* (%) | AVG RVIS (percentile) | scored *de novo* / all *de novos* (%) | AVG RVIS (percentile) | scored *de novo* / all *de novos* (%) | AVG RVIS (percentile) |
| **AUTISM**** | **220/226 (97.3%)** | **-0.036 (50.51)** | **556/587 (94.7%)** | **-0.173 (40.60)** | **97/101 (96.0%)** | **-0.630 (16.82)** |
| **EPILEPTIC ENCEPHALOPATHIES** | **51/55 (92.7%)** | **0.251 (69.67)** | **188/191 (98.4%)** | **-0.432 (24.72)** | **34/35 (97.1%)** | **-0.700 (14.82)** |
| **SEVERE ID** | **26/28 (92.9%)** | **0.027 (55.83)** | **100/106 (94.3%)** | **-0.622 (17.36)** | **36/36 (100%)** | **-0.611 (17.51)** |
| **SIB CONTROLS** | **120/130 (92.3%)** | **0.096 (60.71)** | **311/336 (92.6%)** | **-0.056 (48.91)** | **20/22 (90.9%)** | **-0.170 (40.68)** |

*Three ASD trios with identified *de novo* mutations were published by both O’Roak et al. [[10](#_ENREF_10)] and Sanders et al. [[11](#_ENREF_11)]. The duplicate reporting events were considered only once.
^Where multiple *de novos* occurred in the same gene within the same sample (n=9), the most damaging *de novo* mutation effect was considered.
**Included multiplex families (34.5% of trios studied in Neale et al. (2012) had family history of autism - reflecting 6.1% of all *de novo* mutations in the autism group). The three remaining autism cohorts solely sequenced simplex families from the Simons Simplex Collection.
scored *de novo* / all *de novos* (%): The percentage of *de novo* mutations affecting genes that could be assigned a residual variation intolerance score.
AVG RVIS (percentile): Average residual variation intolerance score assigned to the genes affected by the *de novo* mutations, presented as RVIS percentile.
LGD: Likely Gene Disrupting *de novo* mutations.
SIB: Siblings studied as part of the exome sequencing studies.
